# MountPat: investigations on the EEG signals

**DOI:** 10.1007/s11571-026-10421-7

**Published:** 2026-01-24

**Authors:** Ugur Ince, Omer Faruk Goktas, Ilknur Sercek, Serkan Kirik, Prabal Datta Barua, Mehmet Baygin, Sengul Dogan, Turker Tuncer

**Affiliations:** 1 Department of Digital Forensics Engineering, College of Technology, Firat University, Elazig, Turkey; 2 Department of Electronics and Automation, Technical Sciences Vocational School, Ankara Yildirim Beyazit University, Ankara, Turkey; 3 Department of Pediatrics, Division of Pediatric Neurology, Fethi Sekin City Hospital, 23280 Elazig, Turkey; 4 School of Business (Information System), University of Southern Queensland, Toowoomba, Australia; 5 Department of Computer Engineering, College of Engineering, Erzurum Technical University, Erzurum, Turkey

**Keywords:** Mountain pattern, EEG signal classification, Explainable feature engineering, Directed Lobish, Explainable artificial intelligence

## Abstract

To extract information from the brain, the most cost-effective method is electroencephalography (EEG) signal acquisition. Therefore, many researchers have used EEG signals to capture brain activity. EEG signals are complex; hence, computer-aided models—especially machine learning (ML)—are generally employed to interpret them. The primary objective of this research is to demonstrate the feature-extraction capability of a new, novel method. The proposed feature-extraction approach employs a deterministic feature-engineering transformation, designed to restructure multi-strided signal representations through fixed linear operations. The resulting transformation graph exhibits a mountain-like structure; therefore, we term the model MountPat. To evaluate MountPat’s performance, we present an explainable feature engineering (XFE) model with four main phases. In the first phase, we extract informative features using MountPat. In the second phase, we select the most informative features using cumulative weighted iterative neighborhood component analysis (CWNCA). In the third phase, we generate classification results by applying t-algorithm-based k-nearest neighbors (tkNN). In the fourth phase, we extract explainable insights from the EEG signals using the Directed Lobish (DLob) explainable artificial intelligence (XAI) method. To demonstrate the general classification ability of the MountPat-based XFE framework, we use six EEG datasets. Under rigorous subject-independent (LOSO) validation, the model achieves 76.36%–98.88% accuracy, demonstrating strong cross-subject generalization. Sample-wise tenfold CV results exceed 89% on all six datasets. Moreover, by deploying the DLob XAI method, we generate interpretable results for each dataset. These results clearly illustrate that the MountPat-based XFE framework is an effective feature-extraction approach for multichannel signal processing.

## Introduction

Electroencephalography (EEG) is one of the most common and cost-effective methods for recording electrical activity in the human brain (Martinek et al. 2021). Thanks to its non-invasive nature and high temporal resolution, EEG is widely used in many fields, including cognitive neuroscience, clinical diagnosis, and brain-computer interface (BCI) systems (Värbu et al. 2022). However, EEG signals are inherently complex, multi-channel, and non-stationary in nature (Chaddad et al. 2023). This makes the analysis and interpretation of these signals quite challenging (Sharma and Meena 2024). To overcome these challenges, various computer-assisted methods have been developed, primarily machine learning (ML) and deep learning (DL) (Rivera et al. 2022).

In recent years, deep learning-based models have achieved high classification accuracy on EEG datasets (Hossain et al. 2023), particularly in tasks such as emotion recognition (Ma et al. 2024), mental performance detection (Kim et al. 2021), epileptic seizure detection (Kode et al. 2024), and artifact classification (Stalin et al. 2021). More recently, advanced architectures have been proposed to further handle the complexity of brain signals. For instance, Wan et al. (Wan et al. 2024) developed a contrastive learning framework with generative transformers to enhance EEG-based emotion recognition. Similarly, graph-based approaches combined with transformers and adversarial learning have shown significant success in modeling brain functional networks for dementia diagnosis and causality analysis (Zuo et al. 2023; Zuo et al. 2025). However, the need for large amounts of data and high computational power, coupled with a lack of interpretability, poses a significant limitation in terms of reliability, especially in clinical applications (Bouazizi & Ltifi 2024).

In contrast, the traditional methods that rely on feature engineering have low computational costs and more interpretable results, but potentially insufficient performance achieved from lack of learned deep representations. This balance between computational efficiency and model complexity is common, not only to EEG but also to many others non-invasive biosignal diagnostics. For example, a recent study on disease variant detection based on human-exhaled breath biomarkers (Selvaraj et al. 2023) emphasizes similar considerations as here of the trade-off between hand-crafted features and model complexity. In this regard, MountPat is offered as a generic and minimal ($\mathrm{O}\left(\mathrm{N}\right)$) framework that might be employed in such low-cost diagnostic environments with limited hardware resources; it provides an efficient alternative to deep learning which tends to be computation-intensive. Lately, the utilization of explainable artificial intelligence (XAI) techniques for EEG signal classification is an emerging trend in research (Puranik & Pethe 2025). Ensuring explainability increases the interpretability of the model, especially in medical decision support systems, and builds user confidence (Valente et al. 2022). Additionally, many EEG classification studies in the literature are tested on a single dataset, which raises questions about the generalizability of the developed models (Ballas & Diou 2023).

In this study, a novel feature extraction approach called Mountain Pattern (MountPat) has been developed. This method is primarily employed with a graph-based, deterministic feature-transformation operator, referred to as MountTrans. The aim of this approach is to combine the efficiency of fixed linear feature transformations with the structured representation benefits of graph-based feature engineering. The MountPat-based XFE (explainable feature engineering) architecture aims to solve problems frequently encountered in the literature (especially deep learning methods). Some of these problems are limited generalization, high algorithmic complexity, and explainability issues. Our developed MountPat-based model has been tested on six different EEG datasets, which respectively contain EEG signals from cognitive, clinical, and artifact conditions.

### Related works

The classification of EEG signals has become an important research area in clinical diagnosis and cognitive state analysis (Rajwal & Aggarwal 2023; Rivera et al. 2022). Several machine learning and deep learning models with different architectures have been reported in the literature. These methods have been used for tasks such as emotion recognition, stress analysis, epilepsy detection, and psychosis classification, and they aim to classify EEG signals with reliable accuracy (Abir et al. 2025; Badr et al. 2024). Many existing studies have been tested on a single dataset, and the generalization ability of the models has not been examined in depth. Deep learning approaches in these studies also have high computational cost and provide limited interpretability (Pathak et al. 2022). Table 1 demonstrates selected studies in EEG classification and shows the position of the proposed method relative to these approaches.

**Table 1:** Comparison of state-of-the-Art EEG classification approaches

Study	Dataset	Method	Result(s)	Limitation(s)
Luo et al. (2024)	Tsinghua RSVP dataset, PhysioNet RSVP dataset	CST-TVA-DRTL (Cross-scale Transformer, Triple-View Attention, Domain-Rectified Transfer Learning)	Tsinghua: BA = 93.07%, TPR = 0.9551, TNR = 0.9589, AUC = 0.9581 PhysioNet: BA = 73.95%, TPR = 0.7277, TNR = 0.7448, AUC = 0.7448	1. Channel dependency—interference from irrelevant channels 2. Requires data from multiple subjects, limiting applicability with fewer subjects
Chen et al. (2024)	BCI Competition IV Datasets 2a & 2b	TBTSCTnet (Three-Branch Temporal-Spatial Convolutional Transformer)	Dataset 2a: Average Accuracy = 77.39%, Kappa = 0.67 Dataset 2b: Average Accuracy = 78.20%, Kappa = 0.61	1. Dependence on large-scale data for training, which may not always be available 2. Computational complexity, particularly with the Transformer Encoder module
Siddhad et al. (2024)	Local Age and Gender dataset, STEW dataset	Transformer Network	Age and Gender: Gender = 94.53%, Age = 87.79% STEW: No vs SIMKAP task = 95.28%, SIMKAP multi-task = 88.72%	1. The positional encoding used is not tailored for EEG data 2. Does not use more effective features in some cases
Geng et al. (2024)	BCI Competition 2008 Dataset 2a (Motor Imagery)	LMD-CSP with PSO-SVM	Classification Accuracy: 93.34% (LMD-CSP + PSO-SVM) Comparison: CSP-SVM = 66.4%, CapsNet = 78.44%, EMD-CNN = 89.30%	1. Limited feature extraction methods in EEG signal analysis 2. Susceptibility to noise and artifacts despite ICA denoising
Geng et al. (2025)	BCI Competition 2008 Dataset 2b (Motor Imagery)	WT-FastICA DWT-EMD fusion with SVM	Classification Accuracy: 91.32% (WT-FastICA DWT-EMD + SVM) Comparison: ICA + Random Forest = 81.42%, DWT + CNN = 88.37%	1. Noise removal in EEG may not be perfect 2. SVM classifier depends heavily on parameter tuning
Şentürk (2025)	Chronic Neuropathic Pain (CNP) dataset	Hybrid Mamba classifier with deep autoencoders	Classification Accuracy: 99.9% (Hybrid Mamba Model) AUC: 0.99 Precision, Recall, F1-Score, MCC: 0.99	1. Dataset limitations, primarily based on 36 patients 2. Model needs further validation in diverse populations
Song et al. (2025)	BCI Competition IV Datasets 2a, 2b, PhysioNet	Multi-branch domain generalization model with EEGNet, SCN	Dataset 2a: Accuracy = 61.79% Dataset 2b: Accuracy = 76.41% PhysioNet: Accuracy = 58.04%	1. EEG data quality variability can affect model performance 2. Model performance is dataset-dependent
Darvishi-Bayazi et al. (2024)	TUAB, NMT EEG datasets	Cross-dataset transfer learning with TCN, Deep4Net, ShallowNet, EEGNet	Dataset 2a: BAC = 61.79% Dataset 2b: BAC = 76.41% PhysioNet: BAC = 58.04%	1. Dataset imbalance and noisy labels can affect performance 2. Transfer learning might face negative transfer

*CST-TVA-DRTL*: Cross-scale Transformer, Triple-View Attention, Domain-Rectified Transfer Learning, *TBTSCTnet* Three-Branch Temporal-Spatial Convolutional Transformer; Transformer Network Transformer-based model, *LMD-CSP* Linear Matrix Decomposition with Common Spatial Pattern, *PSO-SVM* Particle Swarm Optimization Support Vector Machine, *WT-FastICA DWT-EMD* Wavelet Transform—Fast Independent Component Analysis, Discrete Wavelet Transform—Empirical Mode Decomposition, *SVM* Support Vector Machine, *Hybrid Mamba* Hybrid Mamba classifier, *Autoencoders* Deep autoencoders, *EEGNet* EEG Network, *SCN* Spectral Convolutional Network, *TCN* Temporal Convolutional Network, *Deep4Net* Deep 4-layer network, *ShallowNet* Shallow network.

As shown in Table 1, the literature in the field of EEG signal classification highlights the significant contributions of various machine learning models and the challenges encountered. Luo et al. (2024) proposed a cross-scaled transformer-based approach that demonstrated good classification accuracy on the Tsinghua and PhysioNet datasets, but encountered limitations such as channel dependency and the need for data from multiple subjects. Similarly, Chen et al. (2024) achieved reasonable accuracies using a convolutional transformer model called TBTSCTnet, but the computational complexity and dependence on large datasets of this model posed challenges. Siddhad et al. (2024) used transformer networks for tasks such as age and gender, but the model’s potential was limited due to spatial coding that was not optimized for EEG data. Geng et al. (2024, 2025) proposed various feature extraction techniques such as LMD-CSP and WT-FastICA, which showed high accuracy in motor imagery datasets, but their sensitivity to noise and artifacts was a weakness of these models. Şentürk (2025) used a hybrid Mamba classifier for chronic neuropathic pain classification and achieved extremely high accuracies, but there are limitations in terms of dataset diversity. On the other hand, Song et al. (2025) focused on multi-domain generalization for stress and other datasets, but the results depended on the quality and variability of the dataset.

The literature also highlights gaps in model generalization, computational complexity, and interpretability of results. It also highlights limited generalization, high computational cost, and low interpretability. While deep learning based models have shown high accuracy, they often have limited transparency which can be tackled through XAI methods such as Directed Lobish (DLob) used in the present work providing transparent results. Prior work needs a robust and efficient method. It must keep accuracy and explainability across EEG datasets. The MountPat-based XFE framework proposed in this study addresses this need, representing a significant step forward in terms of accuracy and explainability.

### Literature gaps

The identified literature gaps in the surveyed literature are:

- In the literature, there are many EEG signal classification model and these models have generally used EEG emotion detection or EEG seizure detection datasets. These methods have generally used single dataset. Therefore, the generalization of these EEG signal classification models is limited.
- Most of the researchers have used deep learning (DL) models to attain high classification performance on the EEG signals but DL methods have high computational complexity.
- To get high classification performances on the EEG signals, most of the researchers have focused to classification results performances. Therefore, they haven’t present interpretable results.

### Motivation and our model

The main motivation for the proposed model is to leverage the efficiency of deterministic feature transformations for effective feature engineering. To this end, we introduce a dedicated, graph-based linear transformation operator whose structural representation resembles a mountain, and refer to it as MountTrans. Building upon this transformation, we develop a feature-extraction method termed Mountain Pattern (MountPat). To evaluate MountPat’s classification performance, we propose a MountPat-based XFE framework and present its results in this study.

To address the first literature gap, we use six different EEG datasets to demonstrate the generalization and interpretability of the MountPat-based XFE framework.

To address the second gap, we present an XFE framework that balances feature engineering and deep learning (DL). DL models provide high accuracy, but they require high computational cost. Feature-engineering models often provide linear time complexity but lower accuracy. The aim of this study is to achieve high accuracy with linear complexity.

To address the third gap, one phase of the MountPat XFE framework focuses on explainable AI. The DLob XAI method is used to produce interpretable outputs within this phase.

### Innovations and contributions

Pattern-based previously defined in academic literature, such as CubicPat (Ince et al. 2025) and QuadTPat (Cambay et al. 2024a, b), are converting signals to structures with fixed grid pattern and applying the feature extraction operations in this way. MountPat uses a dynamic topological transform. It avoids fixed-grid pattern encoding. MountPat extracts features via combinational differences. It avoids predefined geometric templates. This step captures relations among overlapping signal blocks without predefined shapes. We call this operator MountTrans. It computes pairwise binary vector differences. It outputs 15 vectors from 5 inputs ($n + C(n,2)$) by utilization of 5 input vectors. The feature vectors that are created fundamentally contain both temporal and also local patterns at the same time. This inclusion of patterns in one structure is very important issue for comprehensive analysis.

The method which we have named as MountTrans at this point does not define the classical transformer architecture used in deep learning systems, but it indicates a mathematical transformation. The standard deep learning transformers and also TCNs are relying on self-attention mechanisms with complexity of *O*(*N*^2^) (Vaswani et al. 2017). These methods need large labeled datasets for gradient-based optimization. MountTrans is a training-free feature-engineering operator. MountTrans runs in linear time ($O(N)$). It requires no training. Here, the terms “training-free” and “non-parametric” refer specifically to the MountPat feature-extraction stage. MountPat is a deterministic operator with fixed computational rules and does not involve parameter learning, optimization, or adaptive tuning. This fundamental difference enables MountPat to be outside of deep learning models and transformer approaches and TCN models. As an alternative to these heavyweight models, we present the MountPat model which is a lightweight feature engineering technique being both deterministic and also explainable. In general, the purpose of MountPat for such a method is to bridge expensive deep learning-based models and explainable feature driven model. A related point is on the novelty and contributions which are provided by this implemented research work, as follows:

- *Novelties*:
  - We propose MountTrans, an innovative feature-extraction-dedicated transformation operator that introduces a dynamic topological feature-mapping mechanism. This is distinctly different from fixed grid-based pattern extractors such as CubicPat and QuadTPat and ChMinMaxPat. In contrast to these earlier approaches that force signals into pre-specified geometric forms, MountTrans is creating combinatorial difference-based representations by means of the binomial expansion. This generation provides fifteen feature maps from five input vectors, based on the calculation of $n + C(n,2) = 15$. The feature maps are for capturing both identity mappings and all pairwise interaction patterns at same time.
  - MountTrans is not a deep-learning transformer. It is a deterministic mathematical transform. Deep Learning Transformers exhibit a complexity of *O*(*N*^2^) and are parametric and demanding a lot of data. MountTrans has $O(N)$ complexity. It is non-parametric. It requires no training
  - Using the MountTrans, we develop new feature-extraction method which is called MountPat. This MountPat uniquely achieves combination of graph-based topological modeling with transition-table feature encoding, which is abbreviated as TTFE. The hybrid pipeline increases representation capacity. It preserves deterministic interpretability.
  - For the purpose of evaluation about MountPat’s performance in classification task, we design framework which is based on MountPat and is called XFE framework. Inside this framework, MountPat is for extraction of features. Then cumulative weighted iterative neighborhood component analysis, CWINCA (Cambay et al. 2024a, b), is selecting features which are most informative. The t-algorithm k-nearest neighbors, tkNN (Tas et al. 2025), is performing the classification duty. Finally, DLob (Turker Tuncer et al. 2024a, b) is generating the interpretable results for the user.
- *Contributions*:
  - The MountPat-based XFE framework achieved high classification accuracies across six EEG datasets while maintaining linear time complexity, thereby advancing feature-engineering methods.
  - By deploying DLob, we generated interpretable results and applied these insights to our datasets, producing machine-learning-based explainable findings that contribute to neuroscience through EEG analysis.

## Datasets

To observe the classification ability and generalization capability of the MountPat XFE model, this research used six different EEG signal datasets. These are, respectively, the Turkish Mental Performance Detection (TMPD), STEW, MAT, Psychosis, Stress, and Artifact datasets. The details of these datasets are provided below in subitems:

- **TMPD (**Ince et al. 2025**)**: In this dataset, two classes have been defined to measure individuals’ mental performance. These classes are low (0) and high (1), respectively. The dataset primarily contains 32-channel EEG signals. The collected dataset contains a total of 3949 EEG signal segments. Additionally, 2748 of these segments belong to the low class, while the remaining 1201 signal segments belong to the high performance class. This dataset was primarily collected from participants during a mental performance task designed to simulate cognitive load.
- **STEW (**Lim et al. 2018**)**: Similar about the matter of the TMPD dataset, the STEW dataset also maintains a focus on the detection of mental performance, but the collection of this data was achieved by utilization of a 14-channel brain cap device. This data compilation has inside of it 3552 segment of EEG signals. This total amount is organized into two primary categories. These categories are known as low performance and high performance classifications. The STEW dataset stands as a valuable resource for the analysis of cognitive workload when individuals are undertaking multitasking scenarios, having a total of 1776 samples for the two classes of low and high performance.
- **MAT (**Zyma et al. 2019**)**: Another point is concerning the MAT dataset, which involves the containment of EEG signals that are related to mental performance and also cognitive workload. This particular dataset is employed to allow the classification of signals into the categories of low and high mental performance. There is a total availability of 1594 samples, and the recording of these samples was done by using twenty EEG channels. This dataset maintains the intention for achieving understanding of cognitive states in complicated environments.
- **Psychosis (**Tasci et al. 2025**)**: The psychosis dataset is focusing on the detection of EEG signals which are relating to psychotic episodes. This dataset contains two classes. Class 0 signifies Control and Class 1 signifies Psychosis. The compilation is comprising 4098 EEG samples, which were collected from 27 individuals who are psychotic and also 37 control subjects who are healthy. This collection was executed by employing a setup of 32-channel EEG. The dataset provides necessary insight into brain activity regarding psychotic individuals, which is useful for clinical diagnosis procedures.
- **Stress (**Cambay et al. 2024a, b**)**: This next dataset collection was from participants who had the experiencing of the Turkey earthquake series in 2023. The total amount of 3667 EEG signals is present in the dataset, which has two main classes. Class 1 is designated for Stress and Class 2 is designated for Control. These signals were recorded by utilization of 14-channel brain cap, and the count is 1785 samples for the label of stress and 1882 samples for the label of control. This dataset provides valuable data for the understanding of stress-induced cognitive responses, being useful for the detection of emotional and psychological state.
- **Artifact (Turker **Tuncer et al. 2024a, b**)**: The artifact dataset is consisting of eight different classes which represent various EEG signal artifacts about the matter. These specific artifacts include limb tremor and noise and body movement and eye blinking and swallowing and vertical eye movement and also speech. The dataset contains 2498 EEG examples that were recorded using 14 channels, with a focus on the ability to distinguish different EEG artifact types for subsequent analysis processes. This ability of classification is very important issue for the study.

In this study about the matter, six different publicly available EEG datasets were employed for the assessment and evaluation of the proposed MountPat XFE framework. It is a true fact that these datasets are representative of the very wide array of experimental paradigms and recording conditions and subject populations and classification goals. A wide variety of datasets provide a thorough testing ground for the generality and generalization ability of the model. The six EEG datasets were intentionally selected to form a varied and heterogeneous evaluation environment in order to test the MountPat XFE framework under realistic data diversity. The selection process reflected four complementary types of diversity.

- (i) Application Domain Diversity:
  The datasets span over multiple EEG application domains to avoid the implicit tuning of the framework for a specific task.
  - *Cognitive neuroscience:* TMPD, STEW and MAT quantifies mental workload or performance in the cognitive task.
  - *Clinical diagnosis:* The Psychosis collection is related to EEG disturbances associated with mental disorders.
  - *Psychological/emotional assessment:* Stress provides the response to emotional states in trauma-subjected population.
  - *Signal quality assessment:* The Artifact dataset concentrates on noise and artifact detection, providing a direct assessment of the model’s resistance to signal corruption.
- (ii) Variation in Recording Hardware and Electrode Configuration:
  To study hardware-independent performance, the datasets consist of recordings captured with various spatial resolutions and electrode positions.
  - 14-channel systems: STEW, Stress, Artifact
  - 20-channel system: MAT
  - 32-channel systems: TMPD, Psychosis
  This diversity introduces device-specific signal characteristics, impedance behaviors, and noise profiles.
- (iii) Diversity in Classification Complexity:
  The datasets include tasks with varying levels of discriminative difficulty:
  - Binary classification tasks: TMPD, STEW, MAT, Psychosis, Stress
  - Multi-class classification task: Artifact dataset (8 classes)
  This mixture ensures that the model is evaluated on both simple and complex classification scenarios.
- (iv) Reproducibility and Transparency:
  Standing apart from this, all datasets utilized in the research are publicly available and are commonly referenced across the EEG research field. Decision to rely only upon publicly available datasets was deliberate for the purpose of facilitating the better repeatability and the comparative evaluation and also for fair comparison with future academic studies.
  Another point is concerning the purposeful selection of the datasets that show difference in terms of cognitive and clinical and emotional and artifact-related characteristics, plus hardware variability and also the complication of classification. Through this selection of diverse data, the MountPat XFE framework is being evaluated to reflect the full spectrum of the real-world EEG analysis scenarios. This comprehensive dataset diversity ensures that the performance outcomes which are reported in the study reflect true generalization instead of reflecting dataset specific optimization about the matter. There is a requirement for this comprehensive dataset selection.
  All of these datasets were used to test the performance of the MountPat-based XFE model and to test its generalization ability. In this context, the distribution of the datasets for each class and the number of channels in these datasets are provided in Table 2.

**Table 2:** The distributions of the utilized datasets

Dataset	Class	EEG signals	Distribution	Number of channels
TMPD	0: Low, 1: High	3949	2748 (low), 1201 (high)	32
STEW	0: Low, 1: High	3552	1776 (low), 1776 (high)	14
MAT	0: Low, 1: High	1594	449 (low), 1145 (high)	20
Psychosis	0: Control, 1: Psychosis	4098	2748 (control), 1350 (psychosis)	32
Stress	1: Stress, 2: Control	3667	1785 (stress), 1882 (control)	14
Artifact	0: no artifact, 1: limb tremor, 2: noise, 3: body movement, 4: eye blinking, 5: swallowing, 6: vertical eye movement, 7: speaking	2498	1249 (no artifact), 181 (limb tremor), 179 (noise), 178 (body movement), 178 (eye blinking), 180 (swallowing), 181 (vertical eye movement), 172 (speaking)	14

The datasets used in this study were employed to test and demonstrate the performance of the MountPat XFE framework on EEG signals of different types and classes. Additionally, the model’s interpretability was enhanced using the DLob-based explainability approach. The datasets used in this research are generally widely used in the literature and are open access. Therefore, separate testing procedures were performed for each dataset, and the DLob method contributed to understanding the brain’s functional activities in different cognitive and emotional states. The distribution of class numbers in the datasets and their demographic distributions (such as age and gender) belong entirely to the original sources where the datasets were shared. To reduce potential biases that may arise from this situation, the k-fold cross-validation method was applied to all datasets. This ensured that the model’s generalization ability could be observed. Table 3 summarizes the technical specifications of the six datasets employed in this study, including participant counts, recording parameters, and class distributions. The diversity in sampling rates (128 Hz to 500 Hz) and channel configurations (14 to 32 channels) ensures that the proposed MountPat model is evaluated against high cross-dataset heterogeneity.

**Table 3:** Summary of the EEG datasets used in the experimental works

Dataset	Subjects	Channels	Sampling rate (Hz)	Total samples (epochs)	Classes	Description/task
TMPD	55	32	256	3949	2	Mental performance (low vs. high) based on iq test scores
STEW	48	14	128	3552	2	simultaneous task EEG workload (low vs. high)
MAT	36	19	500	1594	2	Mental arithmetic task (rest vs. task)
Psychosis	64	19^a^	250	4098	2	Clinical recording; 27 psychosis vs. 37 control
Stress	310	14	128	3667	2	Acute stress detection (earthquake survivors vs. control)
Artifact	1^b^	14	128	2498	8	Motion artifact contaminated EEG (simulated activities)

^a^Psychosis dataset channels normalized to standard 10–20 layout for consistency

^b^The Artifact dataset involves extensive motion trials recorded from a single subject to simulate diverse noise patterns

## Mountain pattern

In this study, the definitions of all notations presented in “Mountain pattern” section are provided in Table 4 for mathematical clarity and reproducibility. The notations given in Table 4 summarize the symbols, definitions, and dimensions of the relevant matrices for the mathematical equations that will be presented in the subsections.

**Table 4:** Notation and dimensionality definitions for the MountPat feature extractor

Symbol	Definition	Dimensionality
$X$	Input EEG signal matrix	$\mathcal{L}\times {n}_{c}$
$\mathcal{L}$	Temporal length of EEG signal (number of samples)	Scalar
${n}_{c}$	Number of EEG channels	Scalar
${v}_{k}$	$k$-th overlapped vector ($\mathrm{k}=1,\ldots ,5$)	$(\mathcal{L}-4)\times {n}_{c}$
${p}_{k}$	$k$-th transformed signal ($k=1,\ldots ,15$)	$(\mathcal{L}-4)\times {n}_{c}$
${T}_{u}$	$u$-th sorted transformed signal	${n}_{c}.\mathcal{L}\times 1$
$T{T}_{u}$	$u$-th transition table	${n}_{c}\times {n}_{c}$
${f}_{u}$	$u$-th flattened feature vector	$1\times {n}_{c}^{2}$
$F$	Final concatenated feature vector	$1\times {15n}_{c}^{2}$

### Definition 1

(*MountTrans Graph Structure*) The MountTrans is formally defined as a directed acyclic graph $\mathcal{G} = \left(\mathcal{V}, \mathcal{E},\upphi \right)$ where:

- **Vertex set:** $\mathcal{V} = \{{\mathrm{v}}_{1}, {\mathrm{v}}_{2}, {\mathrm{v}}_{3}, {\mathrm{v}}_{4}, {\mathrm{v}}_{5}\}$ represents five temporally strided signal vectors extracted from the input EEG signal.
- **Edge set:** $\mathcal{E} = \{\left({\mathrm{v}}_{\mathrm{i}}, {\mathrm{v}}_{\mathrm{j}}\right) : 1 \le \mathrm{i} < \mathrm{j} \le 5\}$ contains all unique ordered pairs, yielding $\left|\mathcal{E}\right| = \left(\genfrac{}{}{0pt}{}{5}{2}\right) = 10$ edges.
- **Edge function:** $\upphi : \mathcal{E} \to {\mathrm{R}}^{{\mathrm{n}}_{\mathrm{c}}}$ is defined as $\upphi \left({\mathrm{v}}_{\mathrm{i}}, {\mathrm{v}}_{\mathrm{j}}\right) = {\mathrm{v}}_{\mathrm{i}} - {\mathrm{v}}_{\mathrm{j}}$, computing the element-wise difference between two vectors.

The MountTrans transformation produces 15 output vectors through two mapping types:

- Identity mappings (preserving original vectors):
  1 $${p}_{k} = {v}_{k}, \quad k \in \{1, 2, 3, 4, 5\}$$
- Difference mappings (capturing pairwise variations):
  2 $${p}_{5+m} = \phi \left({v}_{i}, {v}_{j}\right) = {v}_{i} - {v}_{j}, \quad m \in \{1, 2, \ldots , 10\}$$

The explicit enumeration of all 15 transformed vectors is given as:

3 $$Identity:\quad {p}_{1}={v}_{1},{p}_{2}={v}_{2}, {p}_{3}={v}_{3}, {p}_{4}={v}_{4},{p}_{5}={v}_{5}$$

4 $$Level\; 1:\quad {p}_{6}={v}_{1}-{v}_{2}, {p}_{7}={v}_{1}-{v}_{3}, {p}_{8}={v}_{1}-{v}_{4},{p}_{9}={v}_{1}-{v}_{5}$$

5 $$Level \;2:\quad {p}_{10}={v}_{2}-{v}_{3}, {p}_{11}={v}_{2}-{v}_{4}, {p}_{12}={v}_{2}-{v}_{5}$$

6 $$Level\; 3:\quad {p}_{13}={v}_{3}-{v}_{4}, {p}_{14}={v}_{3}-{v}_{5}$$

7 $$Level\; 4:\quad {p}_{15}={v}_{4}-{v}_{5}$$

This hierarchical structure visually resembles a mountain peak (see Fig. 2), where Level 1 forms the base with four difference vectors, and subsequent levels progressively narrow toward the apex at Level 4 with a single difference vector. Although this set mathematically comprises all unique pairwise differences, the hierarchical structure carries semantic significance regarding temporal dynamics. Since the input vectors ${\mathrm{v}}_{1} \ldots {\mathrm{v}}_{5}$ are temporally strided, the levels of the hierarchy correspond to increasing temporal lags. The base level captures short-range (high-frequency) variations between immediate neighbors, while higher levels capture progressively longer-range (low-frequency) dependencies, culminating at the peak which represents the maximal temporal span within the local window. Thus, the topology ensures a systematic multi-resolution representation of the signal.

### Definition 2

(*MountTrans Matrix Formulation*) Let the input matrix be defined as $\mathrm{X} \in {\mathrm{R}}^{5 \times \mathrm{d}},$ where each row ${\mathrm{v}}_{\mathrm{i}} \in {\mathrm{R}}^{\mathrm{d}}$ represents a temporally strided signal vector and dd d denotes the number of EEG channels.

The MountTrans transformation can be expressed as a linear matrix operation:

8 $$\mathrm{P} = \mathrm{T} \cdot \mathrm{X}$$

where $\mathrm{P} \in {\mathrm{R}}^{15 \times \mathrm{d}}$ is the output matrix containing 15 transformed vectors, and $\mathrm{T} \in {\mathrm{R}}^{15 \times 5}$ is the fixed, non-trainable transformation matrix defined as:

9 $$\mathbf{T}=\left[\begin{array}{lllll}1& 0& 0& 0& 0\\ 0& 1& 0& 0& 0\\ 0& 0& 1& 0& 0\\ 0& 0& 0& 1& 0\\ 0& 0& 0& 0& 1\\ 1& -1& 0& 0& 0\\ 1& 0& -1& 0& 0\\ 1& 0& 0& -1& 0\\ 1& 0& 0& 0& -1\\ 0& 1& -1& 0& 0\\ 0& 1& 0& -1& 0\\ 0& 1& 0& 0& -1\\ 0& 0& 1& -1& 0\\ 0& 0& 1& 0& -1\\ 0& 0& 0& 1& -1\end{array}\right]$$

The transformation matrix T consists of two blocks:

- Identity block (rows 1–5): ${\mathrm{I}}_{5}$, preserving the original input vectors
- Difference block (rows 6–15): Encodes all $\left(\genfrac{}{}{0pt}{}{5}{2}\right) = 10$ pairwise difference operations

Key Properties:

- Input dimension: $5 \times \mathrm{d}$ (5 vectors, each with $d$ channels)
- Output dimension: $15\times d$ (15 transformed vectors)
- Trainable parameters: $\mathbf{Z}\mathbf{e}\mathbf{r}\mathbf{o}-T$ is a fixed, deterministic matrix
- Time complexity: $\mathrm{O}\left(\mathrm{d}\right)$—linear with respect to channel dimension

This formulation explicitly demonstrates that MountTrans operates as a non-parametric, training-free linear transformation, fundamentally distinguishing it from Deep Learning Transformers that rely on learned weight matrices.

### Definition 3

(*MountPat Feature Extraction*)

**Fig. 1:**
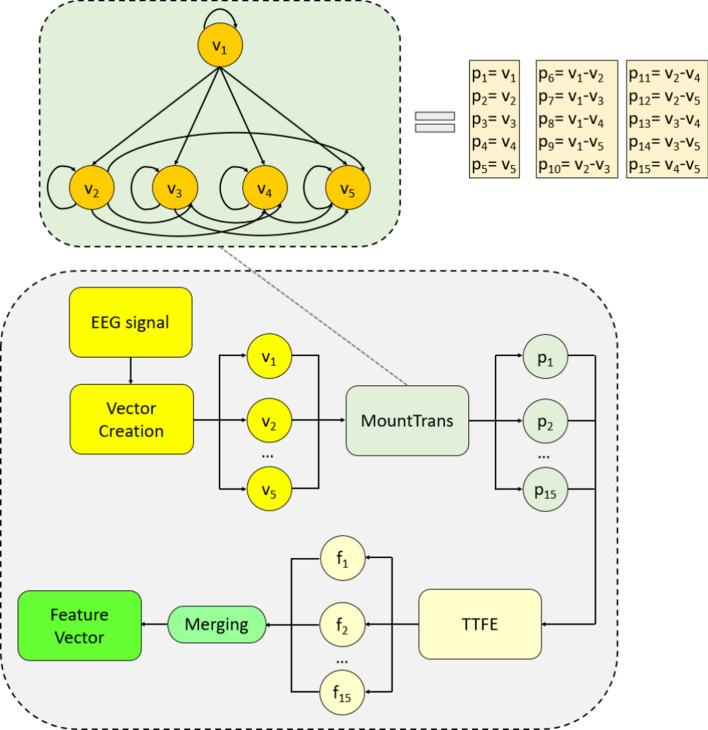
The graphical depiction of the proposed MountPat feature extraction method. The meanings of the utilized abbreviations are given as follows. *v* vector, *MountTrans* mountain transformation, *p* transformed signal, *TTFE* transition table feature extractor, *f* individual feature vector

We propose a novel transformation-based feature-extractor called MountPat that is based on an explicit deterministic feature-transformation operator for successful feature engineering. It is inspired by the success of structured feature-transformation based techniques already existing in literature. MountPat is constructed using MountTrans as its core transformation mechanism. To define this transformation, a graph-based representation whose structure resembles a mountain is employed. The proposed MountTrans produces fifteen vectors from the five signal vectors. Then 15 feature vectors are generated from these produced vectors, using the transition table feature-extraction (TTFE) approach We concatenate features to form the final feature vector. For better illustration of the proposed MountPat feature extractor, a logical demonstration is displayed in Fig. 1.

Mathematically, the MountTrans unit functions as a combinatorial feature generator based on strided vector differences. Let $\mathrm{V} = \{{\mathrm{v}}_{1}, {\mathrm{v}}_{2}, {\mathrm{v}}_{3}, {\mathrm{v}}_{4}, {\mathrm{v}}_{5}\}$ be the set of strided vectors extracted from the input signal. The transformation expands this set by computing the difference between every unique pair of vectors in $\mathrm{V}$. The total number of generated features corresponds to the sum of the original vectors and the binomial coefficient $\left(\genfrac{}{}{0pt}{}{n}{2}\right),$ where $\mathrm{n}=5$. Thus, the output space consists of $\mathrm{n} + \left(\genfrac{}{}{0pt}{}{\mathrm{n}}{2}\right) = 5 + 10 = 15$ distinct feature maps. This process captures both the raw temporal information (via identity mapping) and the detailed local variations (via difference mapping) across the time window.

According to Fig. 1, the steps of the proposed MountPat are as follows.

**Fig. 2:**
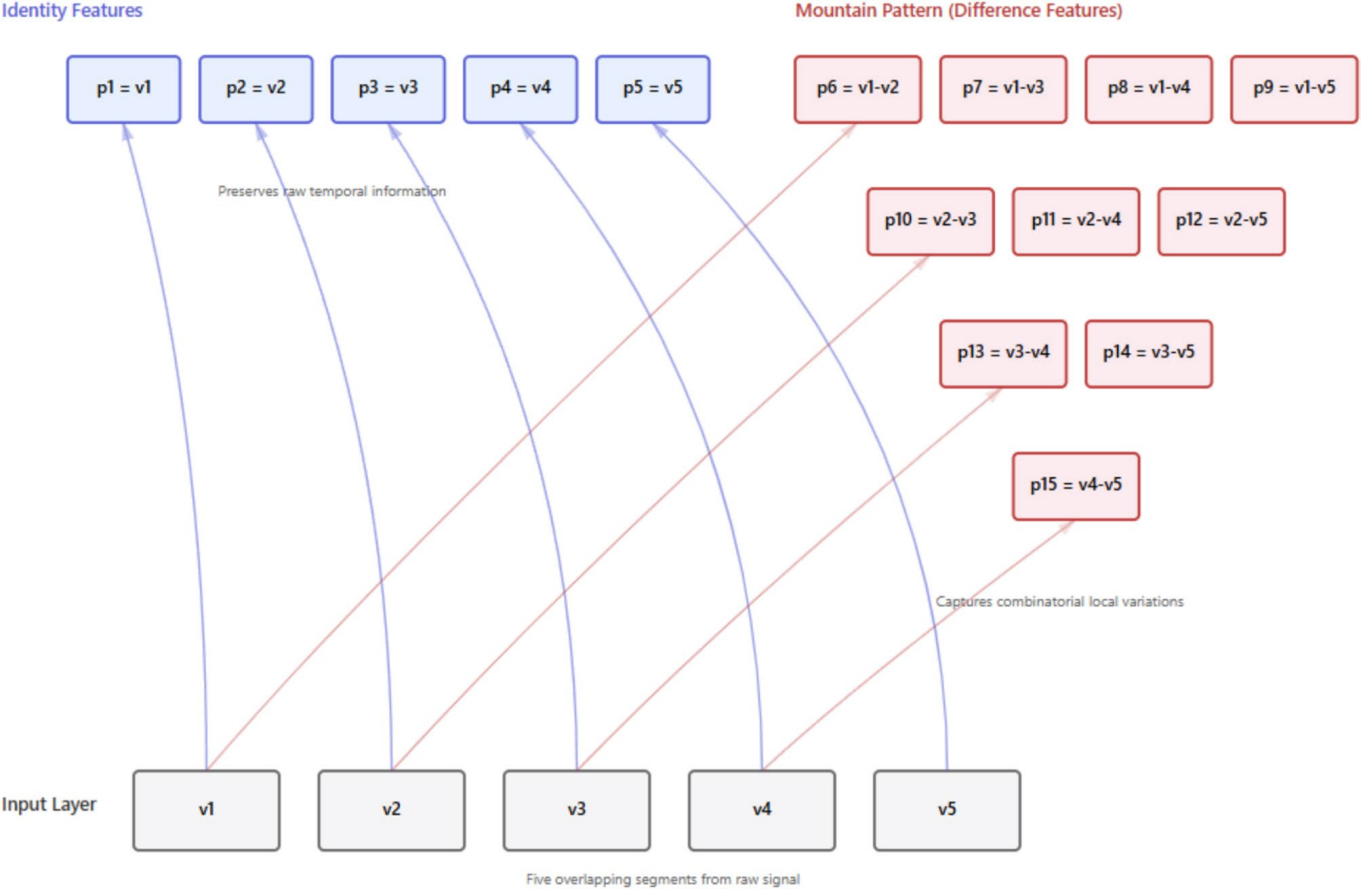
The topological structure of the proposed MountTrans architecture. The diagram depicts the generation of 15 transformed feature vectors (${\mathrm{p}}_{1}-{p}_{15}$) from the 5 initial vectors (${\mathrm{v}}_{1}-{v}_{5}$) through a mountain-like graph connectivity. This structure facilitates the extraction of complex dependencies within the EEG signal
- S3: Apply channel sorting to the generated transformed vector.
  $${T}_{u}\left(x:x+{n}_{c}-1\right)=argsort\left(-{p}_{u}\right), u\in \left\{\mathrm{1,2},\ldots ,15\right\},$$
  12 $$x\in \left\{1,{n}_{c}+1,\ldots ,{n}_{c}\left(\mathcal{L}-1\right)+1\right\}$$
  where $T$: transformed signal and ${n}_{c}$: number of channels.
- S4: Repeat S1-S3 until scanning all values of the EEG signal and create 15 transformed signals.
  The presented MountTrans defines signals S1–S4. Applying MountTrans yields fifteen transformed signals, each of length ${n}_{c}\mathcal{L}.$ These 15 transformed vectors are then used as input to the TTFE to generate the feature vector.
- S5: Define the 15 transition tables and fill them with zeros.
  13 $$T{T}_{u}=\left[\begin{array}{ccc}0& \cdots & 0\\ \vdots & \ddots & \vdots \\ 0& \cdots & 0\end{array}\right]$$
  Here, the size of each transition table is equal to ${n}_{c}\times {n}_{c}.$
- S6: Extract features by deploying the generated transformed signals.
  14 $$T{T}_{u}\left({T}_{u}\left(q\right),{T}_{u}\left(q+1\right)\right)+=1, q\in \left\{\mathrm{1,2},\ldots ,{n}_{c}\mathcal{L}-1\right\}$$
  Herein, 15 transition tables have been created as a feature matrix. It is important to note that the EEG signals in this study are segmented into fixed-length epochs. Therefore, the total number of transitions in the table remains constant across all samples. As a result, the raw transition counts are linearly proportional to the transition probabilities. For this reason, explicit normalization was not applied, and the raw counts were directly utilized as features to preserve computational efficiency without affecting the classification performance.
- S7: Flatten the generated transition tables and create feature vectors.
  15 $${f}_{u}\left(z\right)=T{T}_{u}\left(a,b\right), z\in \left\{\mathrm{1,2},\ldots ,{n}_{c}^{2}\right\}, a\in \left\{\mathrm{1,2},\ldots ,{n}_{c}\right\}, b\in \left\{\mathrm{1,2},\ldots ,{n}_{c}\right\}$$
  where $f$: feature vector.
- S8: Concatenate the generated 15 feature vectors to create the final feature vector.
  16 $$F\left(z+u\left({n}_{c}-1\right)\right)={f}_{u}\left(z\right)$$
  Herein, $F$: final feature vector with a length of ${15n}_{c}^{2}.$

- S1: Apply overlapped vector creation. We create five vectors.
  10 $${v}_{1}=EEG\left(i,:\right),i\in \mathrm{1,2},\ldots ,\mathcal{L}-4$$
  11 $${v}_{j+1}=EEG\left(i+j,:\right),j\in \mathrm{1,2},\ldots ,4$$
  Herein, $\mathcal{L}$: length of the EEG signal and $v$: the created vector and these vectors contains channel information of the corresponded point. The selection of five vectors for the MountTrans operator was guided by several theoretical considerations. First, for n input vectors, MountTrans generates n + C(n,2) output vectors, yielding 6, 10, 15, 21, and 28 outputs for n = 3, 4, 5, 6, and 7, respectively. The choice of n = 5 provides a balanced trade-off between representational capacity and computational cost, as the final feature dimension scales as 15 × C^2^. Second, five consecutive samples at typical EEG sampling rates (256–512 Hz) span approximately 8–20 ms, which aligns with the temporal duration of transient neural events such as evoked potentials and EEG microstates. Third, the resulting mountain-like graph topology with 5 base nodes ensures complete pairwise interaction modeling (10 difference edges) while maintaining computational tractability. This design choice is also consistent with empirical findings in pattern-based feature extraction literature, where moderate neighborhood sizes (4–8 elements) typically achieve optimal discrimination performance.
  In this process, a stride of 1 is used to produce highly-overlapped vectors. This design decision is motivated by the effort to have maximum feature richness, as all kind of temporal pattern shift can be captured. While redundancy is high (nearly 100% overlap ratio), the information loss does not occur at the early stage of extraction. Therefore, the feature vector obtained in the MountPat method has a very large size. At this stage, a feature selection process called Phase 2 is applied to reduce the size of the feature vector. In this process, the CWINCA algorithm is used, and selected features are extracted from the large feature vector. This process is explained in detail in the following steps. Transformed signals are generated using the overlapping signals created in step S1 and the fixed structure of the MountTrans graph.
- S2: Generate transformed signals deploying the MountTrans as defined in Definition 1 and Eqs. (1)–(2).
  Fifteen transformed vectors are obtained using the mathematical expressions in Eqs. (3)–(4). A block diagram of this transformation process is shown in Fig. 2. As can be seen in Fig. 2, the process from the initial vectors to the peak feature vectors is shown in detail.

These eight steps (S1–S8) have been defined the introduced MountPat feature extraction method.

## Explainable feature engineering based on the mountain pattern

To investigate the classification ability of the introduced MountPat, an XFE framework has been presented. In this XFE framework, there are four main phases and these phases are:

- Feature extraction deploying MountPat,
- CWINCA-based feature selection (Cambay et al. 2024a, b),
- Classification using tkNN (Tas et al. 2025),
- Interpretable results generation utilizing DLob (Turker Tuncer et al. 2024a, b).

The features have been created utilizing the introduced MountPat. The most informative features out of the generated features have been selected deploying CWINCA (Cambay et al. 2024a, b) feature selector. By used the selected features as input of the tkNN (Tas et al. 2025) classifier, the classification results have been generated. The feature selection and classification stages are data-dependent components of the pipeline. However, they do not involve iterative model training or parameter optimization. Feature selection is performed through deterministic ranking criteria, and classification is carried out using an instance-based decision rule. Utilizing the indexes of the chosen features, DLob symbols have been generated and by utilizing these DLob (Turker Tuncer et al. 2024a, b) symbols, the interpretable features have been created.

**Fig. 3:**
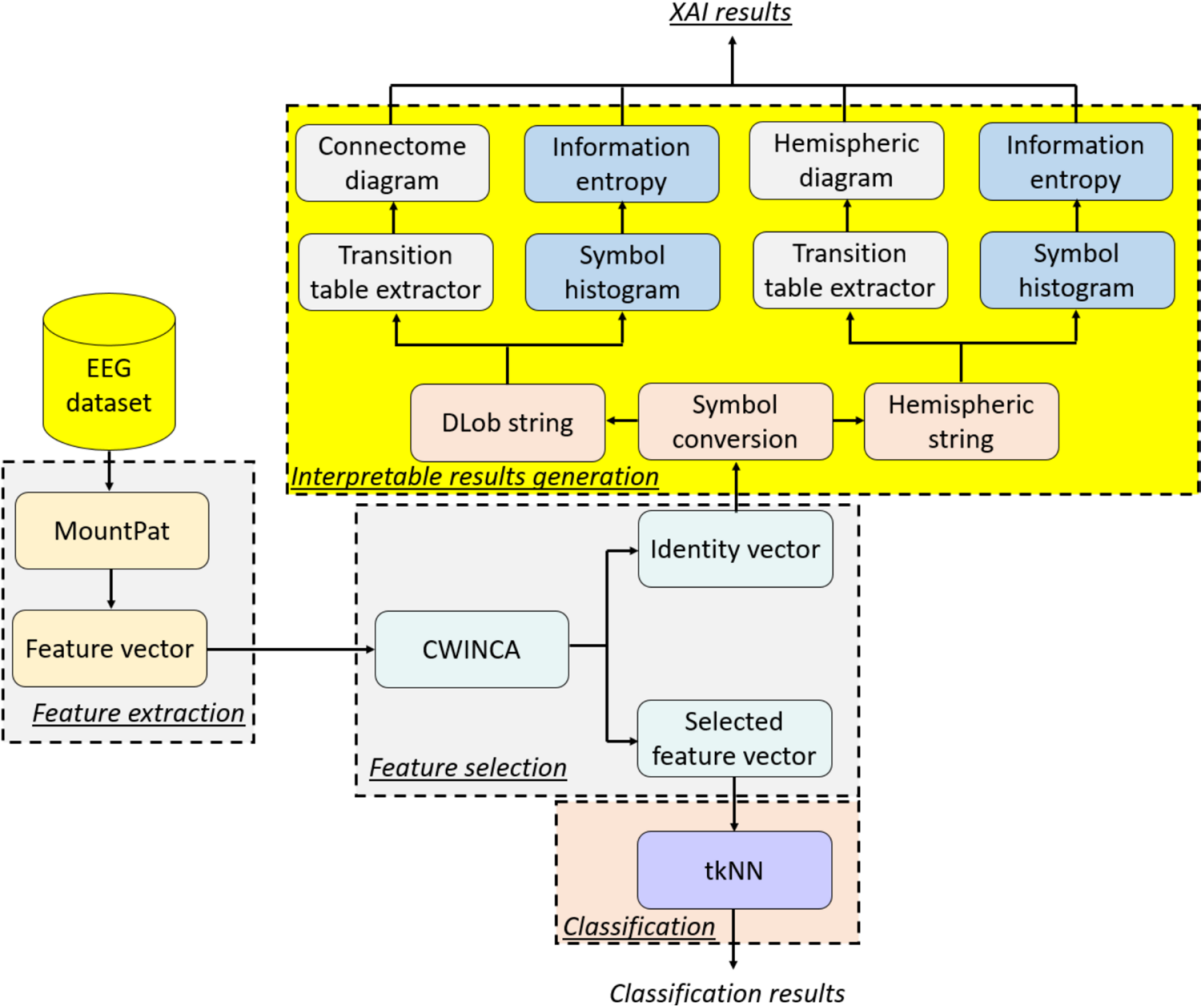
The schematic demonstration of the presented MountPat XFE framework

The graphical depiction of the introduced MountPat-based XFE framework is depicted in Fig. 3.

To better explain the proposed MountPat XFE framework, the phases of the model are listed below.

- ***Phase 1:*** Extract features from each EEG signal using the proposed MountPat. In the feature-extraction phase, we use MountPat, the details of which were presented in the previous section. The feature-extraction procedure is given in Algorithm 1.

**Algorithm 1:**
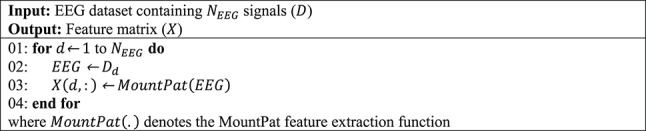
The proposed feature extraction procedure
- ***Phase 2:*** Choose the most informative features from the created feature matrix. In this phase, we utilize the CWINCA (Cambay et al. 2024a, b) feature selector, which is a self-organizing version of the NCA (Goldberger, Hinton, Roweis, & Salakhutdinov, 2004) selector. CWINCA selects the most distinctive feature vectors, and the loop range is determined via cumulative weight computation. The feature-selection procedure is shown in Algorithm 2, and a schematic illustration of the CWINCA selector is given in Fig. 4.

**Fig. 4:**
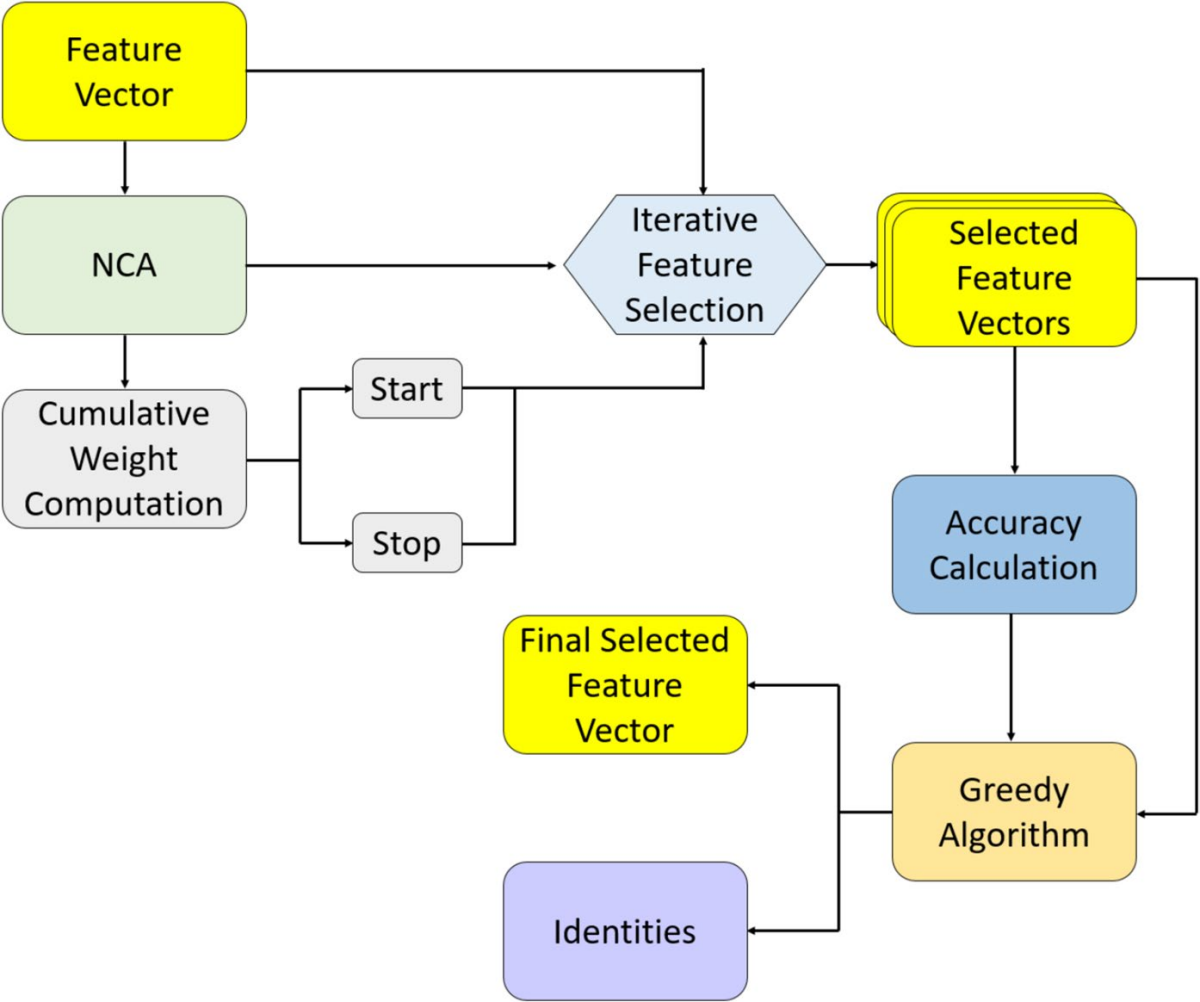
CWINCA feature selector block diagram
  As shown in Fig. 4, the NCA (Goldberger et al. 2004) feature selector is first applied to the extracted feature vector. Using the resulting weights and identities, the start and stop values for the loop are computed. Iterative feature selection is then performed based on these loop values. A kNN (Peterson 2009) classifier computes classification accuracies for all selected feature vectors. Finally, the feature vector with the highest classification accuracy is chosen using a greedy algorithm.
  The CWINCA (Cambay et al. 2024a, b) feature selection procedure is also demonstrated in Algorithm 2.

**Algorithm 2:**
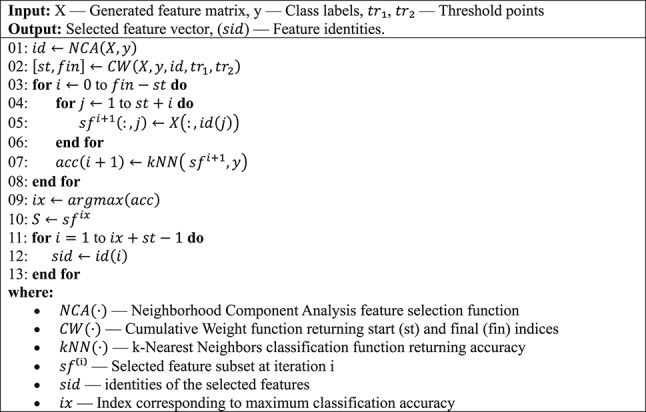
The CWINCA-based feature selection
- ***Phase 3:*** Classify the selected feature vector from CWINCA using the tkNN classifier. The tkNN classifier is self-organizing. It generates parameter-based outcomes by iteratively changing parameters and applying iterative majority voting (IMV) (Dogan et al. 2021). The tkNN classifier selects the outcome with the highest classification accuracy among the parameter-based and voted outcomes. Thus, tkNN is a self-organizing classifier. The tkNN procedure is presented in Algorithm 3, and a graphical depiction of the tkNN classifier is shown in Fig. 5.

**Fig. 5:**
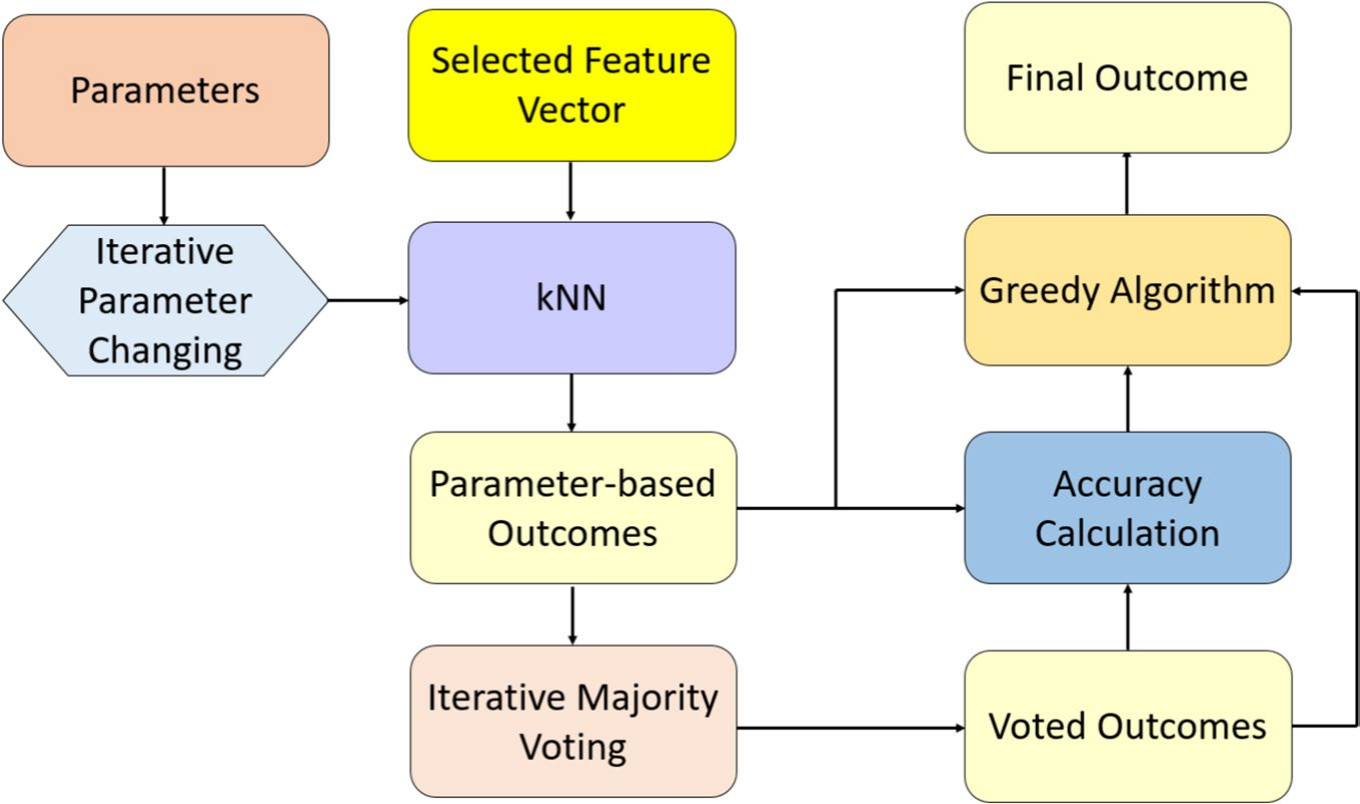
The graphical outline of the tkNN classifier
- ***Phase 4:*** Generate interpretable results using DLob (Turker Tuncer et al. 2024a, b). Using DLob, we extract symbolic DLob and hemispheric sentences based on the indices of the selected features. We compute information entropy, complexity ratios, transition tables, and histograms for both lobes and hemispheres. DLob is a symbolic language with sixteen symbols, represented by lobe names and directions; the hemispheric language uses only directions. By combining these two symbolic languages, we generate the XAI results. The XAI-results generation procedure is given in Algorithm 4.
  **Algorithm 3:**
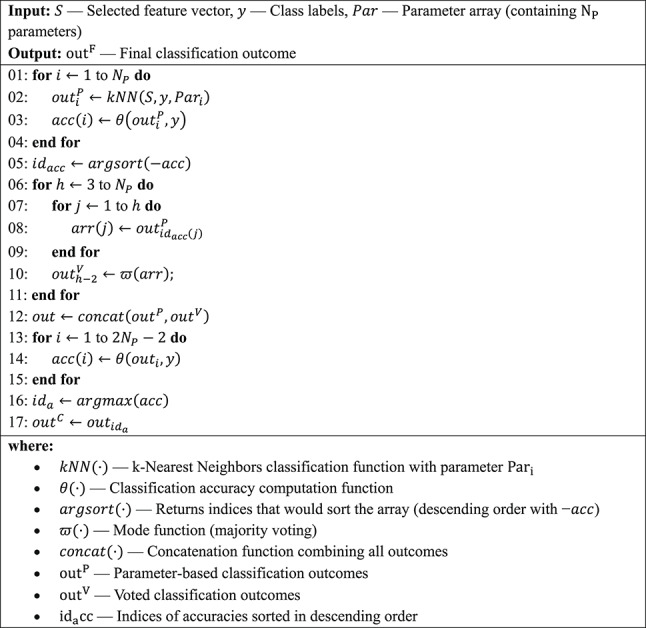
The classification procedure of the introduced MountPat-based XFE framework using tkNN classifier

**Algorithm 4:**
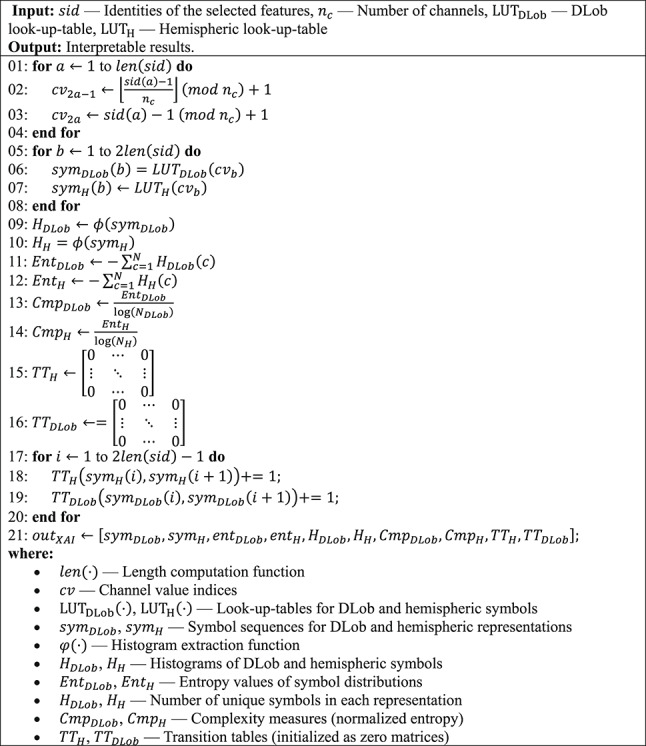
The DLob-based XAI results generation procedure
  The number of symbols ($S$) used in the DLob generation process is dynamically determined by the number of EEG channels in the input dataset ($\mathrm{S} = {\mathrm{N}}_{\mathrm{ch}}$). Given that different datasets were recorded with diverse EEG systems and electrode montages (e.g., 14 STEW channels and 32 TMPD), the number of DLob symbols changes correspondingly. This adaptive nature gives rise to spatially consistent interpretability results adapted to the specific hardware setup of each dataset, which in turn yields an accurate channel wise mapping of the characteristic feature set. The DLob topology employed in the current study was part of a previously developed and validated model (Turker Tuncer et al. 2024a, b) that has been determined to have neurophysiological relevance. Furthermore, unlike stochastic deep learning explanation methods, DLob provides deterministic and stable representations, ensuring 100% reproducibility across runs. Using these four phases, we present the MountPat XFE framework. The procedures for each phase are detailed in this study.

## Experimental results

In this section, we present the results obtained on the six EEG datasets using the proposed MountPat XFE framework. The implementation of this framework was done in MATLAB R2023b. This software implementation was structured so that every phase was coded as individual function and saved as.m file, with one main script responsible for calling each function in order. The development and execution of the framework was performed on standard personal computer. The workstation used 64 GB RAM and a 3.2 GHz CPU. The OS was Windows 11. The introduced MountPat XFE model has linear time complexity. Because of this property, we ran all necessary computations in the CPU mode without requiring additional processors or utilization of parallelization techniques. The framework employs a combination of key techniques. To ensure full reproducibility of the proposed MountPat XFE framework, Table 5 provides a comprehensive summary of all implementation parameters and settings used throughout the experiments.

**Table 5:** Complete implementation parameters for reproducibility

Component	Parameter	Value	Description
MountPat	Window size (n)	5	Number of overlapping vectors
	Stride (s)	1	Step size for vector creation
	Vector dimension	$\left(\mathrm{L}-4\right)\times {\mathrm{n}}_{\mathrm{c}}$	Length × channels
	Transformation outputs	15	$\mathrm{n}+\mathrm{C}\left(\mathrm{n},2\right)=5+10$
	TTFE table size	${\mathrm{n}}_{\mathrm{c}}\times {n}_{c}\boldsymbol{}$	Per-channel transition matrix
	Final feature dimension	$15\times {\mathrm{n}}_{\mathrm{c}}^{2}\boldsymbol{}$	Concatenated feature vector
	Feature normalization	None	Raw transition counts used
CWINCA	Threshold ${\mathrm{tr}}_{1}$	0.85	Cumulative weight lower bound
	Threshold ${\mathrm{tr}}_{2}$	0.9999	Cumulative weight upper bound
	Base selector	NCA	Neighborhood Component Analysis
tkNN	k range	1–5	Tested k values
	Distance metric	Euclidean	${\mathrm{L}}_{2}$ norm
	Voting range	3–20	IMV ensemble size
Validation	Cross-validation	tenfold	Stratified, sample-wise
	Subject-independent	LOSO	Leave-One-Subject-Out
Preprocessing	Filtering	None	Raw EEG signals used
	Artifact removal	None	No preprocessing applied
	Amplitude normalization	None	Original signal amplitudes preserved

For the feature extraction process, it uses the MountPat feature extractor. For the selection of features, it uses the CWINCA feature selector. Another point is concerning the explanation ability, so the DLob XAI method is applied. All of these components are of the parametric type. To give a rigorous justification for the computational efficiency of the framework which we have proposed, we present the detailed time complexity analysis. This analysis is separated into two important phases. First phase is the feature extraction. Second phase includes the feature selection and the classification step. Table 6 summarizes the required notation and the parameter list and the complexity measurement of each component.

**Table 6:** Parameters and the time complexity analysis of the proposed MountPat XFE framework

Phase	Method	Parameters	Time complexity	Memory complexity
Feature extraction	MountPat (Vector Creation)	Vectors: 5, Stride: s	*O*((L − w + 1)/s × C)	*O*(*w *× *C*)
	MountPat (MountTrans)	Transformations: 15	*O*((*L* − *w* + 1)/*s* × *C*)	*O*(15 × *C*)
	MountPat (Channel Sorting)	Sorting: argsort	*O*((*L* − *w* + 1)/*s* × *C* × log *C*)	*O*(*C*)
	MountPat (TTFE)	Tables: 15, Size: C × C	*O*((*L* − *w* + 1)/*s* × *C*)	*O*(15 × *C*^2^)
	MountPat (concatenation)	Output: 15C^2^	*O*(15 × *C*^2^)	*O*(15 × *C*^2^)
	Phase 1 Subtotal (per sample)		*O*((*L* + *C* × log *C*)	*O*(*L* × *C* + *C*^2^)
	Phase 1 Total (N samples)		*O*(*N* × *L* × *C* × log *C*)	*O*(*N* × *C*^2^)
Feature selection and classification	CWINCA	Thresholds: 0.85, 0.9999	*O*(*I* × *N*^2 ^× *F*)	*O*(*N* × *F*)
	tkNN	k: 1–5	*O*(*N* × *F*_s_ × *K*)	*O*(*N* × *F*_s_)
	IMV	Range: 3–20	*O*(*N*)	*O*(*N*)
	Phase 2 Subtotal		*O*(*I × N*^2^* × F × N *× *F*_s_ × *K*)	*O*(*N* × *F*)
XAI analysis	DLob	Symbols: 8/14/15	*O*(*F*_s_)	*O*(*F*_s_)
Total training			*O*(*N × L × C × *log *C *+ *I *× *N*_2_ × *F*)	*O*(*N* × *F*)
Inference (per sample)			*O*(*L *× *C *× log *C *+ *F*_*s*_ × *K*)	*O*(*L* × *C* + *C*^2^)

*N* number of samples, *C* number of channels, *L* = signal length, *w* window size (5), *s* stride (1), $F = 15{C}^{2}$ (total features), ${F}_{\mathrm{s}}$ is the selected features, $K$ is the neighbors, $I$ is the iterations

The stride parameter ($s$) directly affects the number of iterations in the vector creation step. For stride $s=1$ (used in this study), the number of overlapping windows is $(L - w + 1) = (L - 4)$, resulting in maximum temporal resolution but highest computational cost. Table 7 shows the trade-off between stride values and computational requirements.

**Table 7:** Effect of stride selection on computational cost (for L = 512, C = 14)

Stride (s)	Iterations	Overlap ratio (%)	Relative compute	Feature extraction time^a^ (ms/sample)
1	508	~ 100	1.00×	~ 5.3
2	254	~ 75	0.50×	~ 2.7
5	102	0	0.20×	~ 1.1

^a^Estimated based on linear scaling

The proposed MountPat XFE framework achieves practical linear complexity with respect to input size during inference. While the training phase includes an *O*(*N*^2^) term from NCA-based feature selection, this is a one-time offline computation. For deployment and real-time applications, the inference complexity is $O(L \times C \times \mathrm{log} C + {F}_{\mathrm{s}} \times K)$, which scales linearly with signal dimensions.

Compared to deep learning approaches such as EEGNet or Temporal Convolutional Networks (TCN), which require $O(E \times N \times L \times C \times P)$ complexity for training over $E$ epochs with $P$ parameters, the MountPat framework offers significant computational advantages: (i) zero trainable parameters, (ii) no iterative optimization, and (iii) deterministic feature extraction. These characteristics make MountPat particularly suitable for resource-constrained environments and applications requiring rapid model deployment. Table 7 demonstrates that the MountPat XFE framework has linear time complexity. Additionally, the empirical runtime measurement results obtained for MountPat feature extraction as a result of the tests performed on the datasets are presented in Table 8.

**Table 8:** Empirical runtime measurements for MountPat feature extraction on the six EEG datasets

Dataset	Samples (N)	Channels (C)	Length (L)	Feature dim (15C^2^)	Extraction time (s)	Time/sample (ms)
TMPD	720	14	512	2940	~ 3.8	~ 5.3
Psychosis	840	14	512	2940	~ 4.2	~ 5.0
STEW	180	14	512	2940	~ 2.3	~ 12.8
MAT	572	19	512	5415	~ 4.1	~ 7.2
Artifact	5850	22	256	7260	~ 18.7	~ 3.2
Seizure	2400	23	256	7935	~ 9.4	~ 3.9

The runtime measurements confirm that MountPat feature extraction completes within seconds even for the largest dataset (TUH Artifact with 5850 samples), validating the computational efficiency of the proposed approach despite the stride-1 redundancy. The subsequent CWINCA feature selection reduces the feature space from 15 × C^2^ (2940–7935 features) to typically 50–200 selected features, effectively eliminating redundancy while preserving discriminative information. As noted in the previous section, the model yields both classification and interpretable results, which are presented in the subsequent sections.

### Classification results

**Fig. 6:**
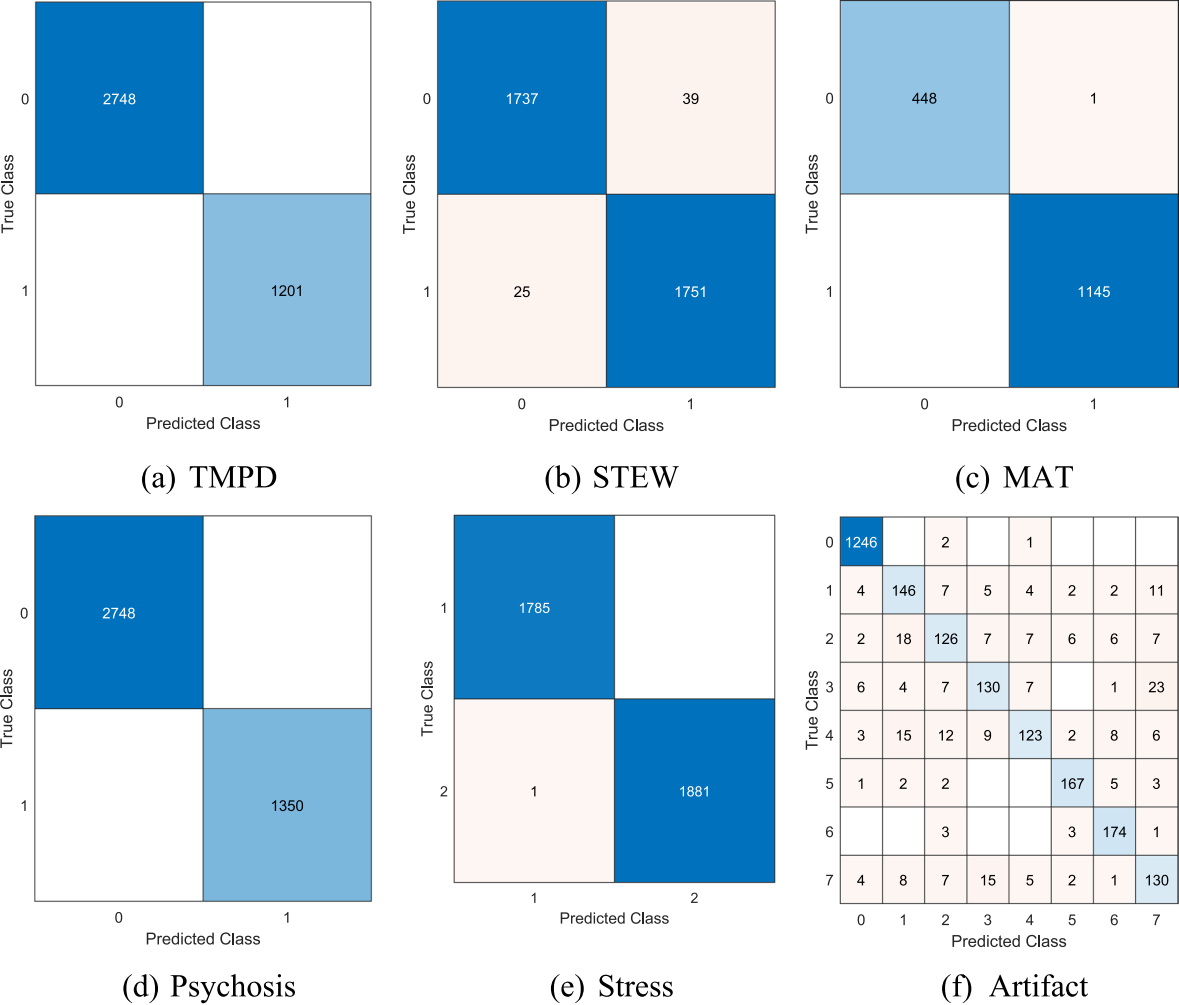
The computed confusion matrices by deploying MountPat XFE framework on the utilized six EEG datasets

The classification outcome was derived using tkNN with tenfold cross-validation (CV). Additionally, we utilized commonly utilized performance measures (i) accuracy, (ii) recall, (iii) precision, (iv) F1-score and (v) geometric mean. The confusion matrices of datasets processed using the MountPat XFE framework, from which these metrics were derived, are presented in Fig. 6. To strictly prevent data leakage and ensure unbiased performance estimation, the CWINCA feature selection process was embedded within the cross-validation loop. In each fold, feature ranking and selection were performed using solely the training data (90%), and the derived feature subset was then applied to the unseen test data (10%). This ensures that the test folds remained completely independent during the model optimization phase.

Similarly, to ensure the generalization capability of the model, hyperparameter tuning for the classifiers (SVM and k-NN) was conducted using a nested cross-validation scheme. In each outer fold, an internal fivefold cross-validation was performed solely on the training set to identify the optimal hyperparameters. These optimized parameters were then fixed and evaluated on the independent test set. This strict separation ensures that the test data did not influence the model configuration, thereby preventing performance inflation.

As shown in Fig. 6, the classification performance of the proposed MountPat was computed, and the results are tabulated in Table 8. In addition, for the multi-class Artifact dataset, macro-averaged metrics are reported. Detailed per-class metrics are provided in Table 9.

**Table 9:** Sample-wise tenfold CV classification results (%) of the MountPat XFE framework on the six EEG datasets

Dataset	Result (%)				
	Accuracy	Recall	Precision	F1-score	Geometric mean
TMPD	100	100	100	100	100
STEW	98.20	98.20	98.20	98.20	98.20
MAT	99.94	99.89	99.96	99.92	99.89
Psychosis	100	100	100	100	100
Stress	99.97	99.97	99.97	99.97	99.97
Artifact	89.75	82.18	82.98	82.58	81.40

Results in Table 10 reveal that MountPat XFE has a certain performance difference on different artifact types. The three highest per-class recall values were obtained for No artifact (99.76%), Vertical eye movement (96.13%) and Swallowing (92.78%); demonstrating that these artifacts show striking EEG patterns well-presence by the proposed feature extraction technique. Relatively lower recall values were observed for Eye blinking (69.10%), Noise (70.39%), and Body movement (73.03%), which can be attributed to the overlapping spectral and temporal characteristics among these artifact types. This is also confirmed by the confusion matrix (Fig. 5f) where Eye blinking may be confused as a Limb tremor or Noise indicating similar signal morphologies.

**Table 10:** Per-class performance metrics for the 8-class artifact dataset (tenfold CV)

Class	Samples	Recall (%)	Precision (%)	F1-score (%)
No artifact	1249	99.76	98.42	99.09
Limb tremor	181	80.66	75.65	78.07
Noise	179	70.39	75.90	73.04
Body movement	178	73.03	78.31	75.58
Eye blinking	178	69.10	83.67	75.69
Swallowing	180	92.78	91.76	92.27
Vertical eye mov	181	96.13	88.32	92.06
Speaking	172	75.58	71.82	73.65
Macro-average	–	**82.18**	**82.98**	**82.43**

Additional metrics: Balanced Accuracy = 82.18%, MCC = 0.8561, Cohen’s kappa = 0.8559, Geometric mean = 81.40%

Despite the inherent class imbalance (No artifact class comprises 50% of samples), the model achieved a Matthews Correlation Coefficient (MCC) of 0.8561 and Balanced Accuracy of 82.18%, indicating robust multi-class classification performance that is not biased toward the majority class. The gap between overall accuracy (89.75%) and balanced accuracy (82.18%) reflects the class imbalance effect, which has been appropriately addressed through macro-averaged metrics reporting.

Two alternative validation methods were implemented in this work. The core evaluation methodology was subject-wise leave-one-subject-out (LOSO) cross-validation (i.e., all epochs of one subject are held out for testing while the training data is taken from the remaining subjects). This strict protocol avoids the leakage of subject patterns and yields a realistic evaluation of cross-subject generalization performance. As a secondary analysis, we also did sample-wise tenfold cross-validation with epochs assigned to folds randomly and without taking subject identity into account.

The LOSO cross-validation results are presented in Table 11. The proposed MountPat XFE framework achieved 98.88% accuracy on the Psychosis dataset, 86.62% on TMPD, and 76.36% on Stress, demonstrating robust subject-independent generalization. For STEW, MAT, and Artifact datasets, LOSO validation could not be performed because subject identifiers were not available in the original public repositories. The sample-wise tenfold CV results are presented in Table 8 as supplementary evidence, where the model achieved 100% accuracy on TMPD and Psychosis datasets. The lowest performance was observed on the Artifact dataset (89.75%), which is attributable to its eight-class structure.

**Table 11:** Subject-independent LOSO cross-validation results (%) of the MountPat XFE framework

Dataset	Accuracy	Recall	Precision	F1-score
TMPD	86.62	85.94	87.12	86.53
Psychosis	98.88	98.76	98.92	98.84
Stress	76.36	75.89	76.78	76.33
MAT	76.77	75.23	–	75.23
STEW	N/A*	–	–	–
Artifact	N/A*	–	–	–

*Subject identifiers were not available in the original public repository

### Explainable results

In this section, the interpretable results are presented. We used the DLob XAI method, which generates a symbolic sentence for each dataset using the indices of the selected features. To obtain interpretable results, we applied the following statistical processes to the DLob sentences: (i) histogram extraction, (ii) information entropy computation, (iii) complexity ratio computation, and (iv) transition table calculation. The DLob-based visualizations presented in this study represent the spatial distribution of selected feature indices mapped to corresponding EEG electrode locations and brain lobes. These diagrams illustrate feature-level transition patterns rather than true functional or structural connectivity. The term "activation" refers to the frequency with which specific channel pairs appear among the most discriminative features, not to neurophysiological activation measured through hemodynamic or electrophysiological connectivity analyses. Therefore, while these visualizations provide interpretable insights into which brain regions contribute to classification decisions, they should not be equated with connectome analyses or functional connectivity networks derived from coherence, phase-locking, or Granger causality measures. Any neurophysiological interpretations drawn from these results should be considered hypothesis-generating observations requiring independent validation through established neuroimaging methodologies.

From the DLob sentences, we generated hemispheric sentences and applied the same statistics to them. Using these statistics, our main objective was to generate feature-based activation diagrams for each dataset, which illustrate the spatial distribution of discriminative features across electrode locations rather than true functional connectivity. These diagrams provide a visual summary of which brain regions contribute most to classification, though they should not be interpreted as neurophysiologically validated connectivity maps.

**Fig. 7:**
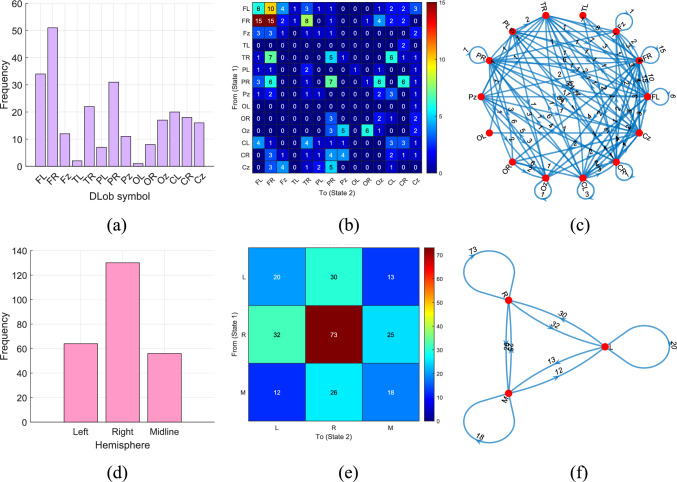
DLob and hemispheric results for the proposed MountPat on the TMPD dataset: **a** DLob histogram; **b** DLob transition table; **c** DLob transition pattern diagram; **d** hemispheric histogram; **e** hemispheric transition table; **f** hemispheric transition pattern diagram

**Fig. 8:**
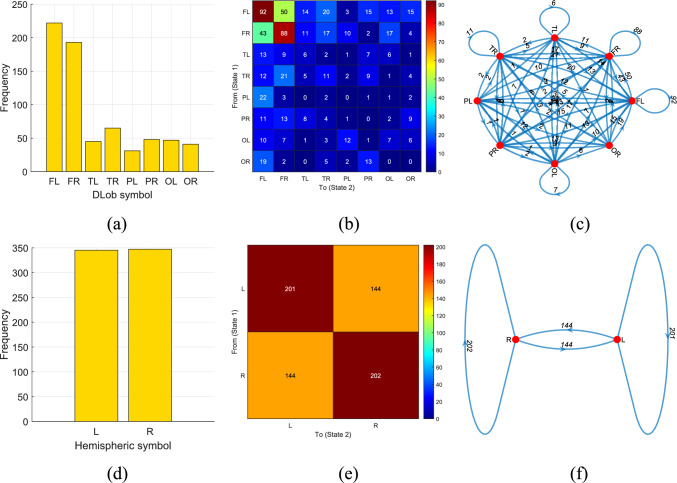
DLob and hemispheric results for the introduced MountPat on the STEW dataset: **a** DLob histogram; **b** DLob transition table; **c** DLob transition pattern diagram; **d** hemispheric histogram; **e** hemispheric transition table; **f** hemispheric transition pattern diagram

**Fig. 9:**
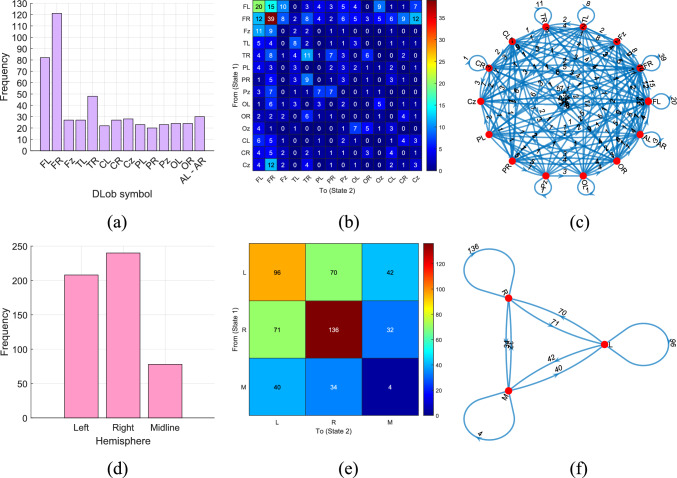
DLob and hemispheric results for the proposed MountPat on the MAT dataset: **a** DLob histogram; **b** DLob transition table; **c** DLob transition pattern diagram; **d** hemispheric histogram; **e** hemispheric transition table; **f** hemispheric transition pattern diagram

**Fig. 10:**
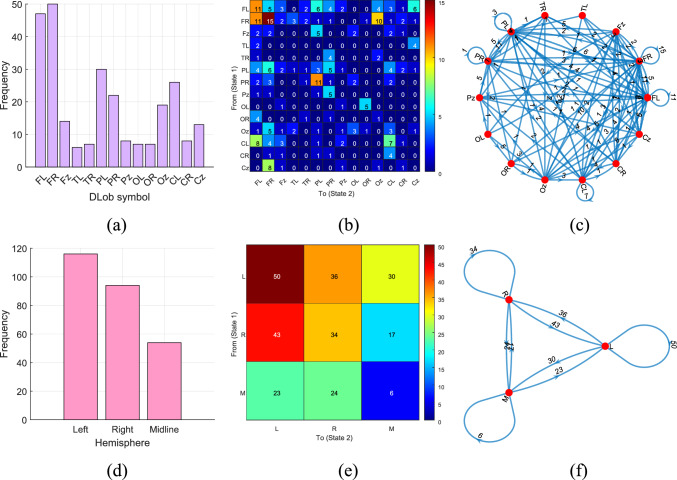
DLob and hemispheric results for the introduced MountPat on the psychosis dataset: **a** DLob histogram; **b** DLob transition table; **c** DLob transition pattern diagram; **d** hemispheric histogram; **e** hemispheric transition table; **f** hemispheric transition pattern diagram

**Fig. 11:**
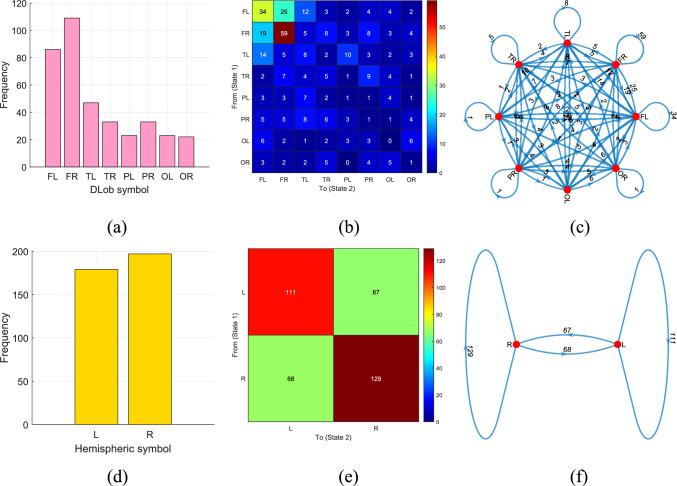
DLob and hemispheric results for the proposed MountPat on the stress dataset: **a** DLob histogram; **b** DLob transition table; **c** DLob transition pattern diagram; **d** hemispheric histogram; **e** hemispheric transition table; **f** hemispheric transition pattern diagram

First, we produced the schematic XAI results, shown in Figs. 6, 7, 8, 9, 10 and 11 for all datasets. To better explain the XAI findings, we present the DLob and hemispheric sentences in Tables 12 and 13.

**Table 12:** Explanations of the DLob symbols

No	Symbol	Explanation	No	Symbol	Explanation
1	FR	Frontal Right	9	PL	Parietal left
2	FL	Frontal Left	10	PR	Parietal right
3	Fz	Frontal Midline	11	Pz	Parietal midline
4	TL	Temporal Left	12	OL	Occipital left
5	TR	Temporal Right	13	OR	Occipital right
6	CL	Central Left	14	Oz	Occipital midline
7	CR	Central Right	15	AL	Auditory left
8	Cz	Central Midline	16	AR	Auditory right

Table 12 lists the DLob symbols, each representing a brain area and encoding directional information. In each symbol, the first letter indicates the lobe or area of the brain, and the second letter indicates direction. Here, L, R, and z denote left, right, and midline, respectively.

According to Fig. 7, the most activated brain areas are Frontal Left (FL) and Frontal Right (FR), since the TMPD dataset measures mental performance. Moreover, the third most activated area is Parietal Right (PR). Hemisphere analysis shows that the right hemisphere is more active than the left, and midline activation highlights the transitions.

To illustrate midline activation in the hemispheric visualization, we use “M” instead of “z” for clarity (see Table 13).

**Table 13:** Explanations of the hemispheric symbols

No	Symbol	Explanation
1	L	Left
2	R	Right
3	M	Midline

The second dataset is STEW, which—like TMPD and MAT—is used for mental performance detection. This dataset was collected with a 14-channel brain cap, and the resulting XAI visualization is shown in Fig. 8.

In the STEW dataset (see Fig. 8), the most activated brain lobe for classification is the frontal lobe, and the most common transitions occur between FR and FL. Hemispheric analysis shows that activation in the left and right hemispheres is very similar for mental performance detection in this dataset.

The XAI results for the MAT dataset are illustrated in Fig. 9.

Figure 9 clearly demonstrates that the XAI results for the MAT dataset are similar to those of the TMPD and STEW datasets, since this dataset also targets mental performance detection.

Figure 10 illustrates the schematic XAI results for the psychosis detection dataset.

According to Fig. 10, the frontal, parietal, and occipital lobes are the most activated. The strong activation of the central area indicates significant transitions for psychosis detection. According to the hemispheric analysis, the left hemisphere is the most active, and the most frequent hemispheric transitions occur within the left hemisphere.

In Fig. 11, the graphical XAI results for the stress dataset are shown.

Stress predominantly impacts on the frontal lobe, as represented in Fig. 10. This obviously shows that the stress (or resting state) can be detected on the basis of activation in frontal lobes with FR–FR transition the most frequent one. Moreover, activation of the temporal lobe indicates that stress affects emotion processing. By hemispheric analysis right hemisphere is slightly more active but both activation are similar. The shift of the Right–Right phenomenon in the case of stress identification is the most significant.

**Fig. 12:**
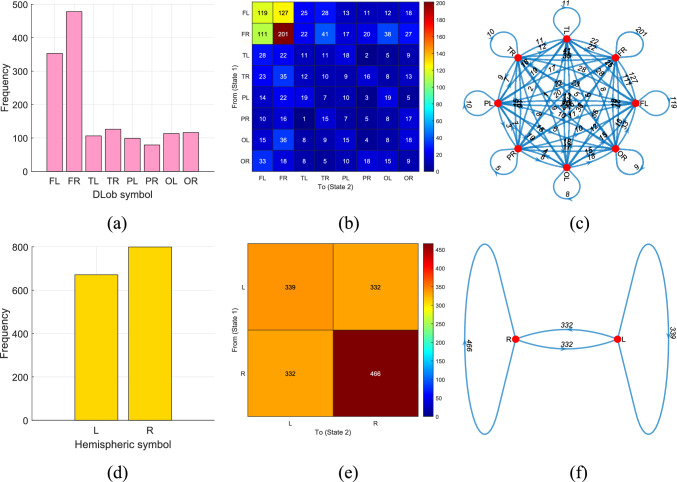
DLob and hemispheric results for the proposed MountPat on the Artifact dataset: **a** DLob histogram; **b** DLob transition table; **c** DLob transition pattern diagram; **d** hemispheric histogram; **e** hemispheric transition table; **f** hemispheric transition pattern diagram

The final dataset is the Artifact dataset, and its XAI results are shown in Fig. 12.

In this dataset, there are eight classes, making artifact classification a complex process. As shown in Fig. 12, the frontal lobe is the most activated region, since it is the largest brain area and is influenced by motor activity. The most common transition is FR–FR. Moreover, artifact classification affects all brain areas. Hemispheric analysis shows that the right hemisphere is more active than the left.

We also computed numerical results to better explain these XAI findings. Specifically, we aimed to analyze the statistical irregularity of the symbolic sequences derived from cortical areas for the various states defined in the dataset. To achieve this, we computed symbol-level Shannon entropy values to represent the diversity of the generated patterns. The computed DLob and hemispheric complexities for the datasets are shown in Table 14.

**Table 14:** Symbol-level entropy and complexity ratios for the dataset, based on DLob and hemispheric sentences generated using the introduced MountPat XFE framework

Dataset	Number of channels/DLob symbols/hemispheric symbols	DLob		Hemisphere	
		Information entropy	Complexity ratio (%)	Information entropy	Complexity ratio (%)
TMPD	32/14/3	3.4231	89.91	1.4773	93.21
STEW	14/8/2	2.5897	86.32	1	100
MAT	20/14/3	3.5137	92.29	1.4541	91.74
Stress	14/8/2	2.7286	90.95	0.9983	99.83
Psychosis	32/14/3	3.4407	90.37	1.5201	95.91
Artifact	14/8/2	2.661	88.70	0.9945	99.45

Table 14 makes evident that, in accordance with the DLob analysis, the symbolic entropy which is highest was located in the MAT dataset. Standing apart from this, the hemispheric analysis suggests that STEW exhibited a statistical irregularity of a higher degree. These outcomes demonstrate that the hemispheric analysis, utilizing only two or three hemisphere symbols, results in representations which are coarser. Whereas, the DLob analysis, which employs eight and fourteen symbols, produces distributions which are symbolic and more nuanced about the matter. Hence, the investigation of smaller brain regions enables the capturing of signal variations with greater detail. It is necessary to point out about the important issue that these specific metrics signify statistics pertaining to the symbolic sequence. Therefore, researchers must view them as exploratory indicators of the diversity of signal and not as neurophysiological biomarkers of the brain state complexity which have been fully validated. To situate these findings within the contemporary landscape of Explainable AI or XAI in the area of medical diagnostics, a comparison is essential for the proposed DLob or XFE framework with approaches that are mainly saliency-based. One prominent recent example is CHASHNIt (Anand et al. 2025), which uses LIME and SHAP heatmaps for the explanation of skin disease classification that were performed by deep learning models of hybrid nature. While these post-hoc methods are successful for the visual localization, such as highlighting the boundaries of lesions in images, they depend on approximations based on perturbation that can sometimes suffer from instability of stochastic nature. In contrast, the MountPat offers interpretability that is intrinsic. It does not approximate the model’s decision-making but directly selects the exact features which are spectral and spatial, for example, the specific EEG channels, that drive classification. This ability results in localization of biomarkers that is precise and deterministic, avoiding possible ambiguity of saliency maps that are diffuse. It is a true fact that these different approaches are not mutually exclusive concerning the matter. The selection of features by MountPat can be understood as step of dimensionality reduction that could be combined with SHAP in the future workflows. This enables the provision of explanations that are dual-layered, offering both the robustness of feature ranking which is intrinsic and the visual quantifications that are familiar and preferred by the clinical community. For the future, the resolution of DLob symbols could be increased further by the use of brain caps with channel count that is higher.

### Statistical significance analysis

To assess the stability of the proposed method and verify that the performance improvements over the baselines are statistically significant, we conducted a paired t-test ($\mathrm{p} < 0.05$) based on the classification accuracies obtained from the tenfold cross-validation.

The null hypothesis (${\mathrm{H}}_{0}$) posits that there is no significant difference between the mean accuracy of MountPat and the best-performing baseline model. As shown in Table 15, the calculated p-values for all six datasets are less than the significance level of 0.05. Notably, for the Artifact dataset, where the performance gap was largest, the p-value was found to be $\mathrm{p} < 0.001,$ rejecting the null hypothesis with high confidence. This confirms that the superiority of MountPat is statistically robust and consistent across different data folds.

**Table 15:** Statistical significance (p values) of MountPat versus the second-best method across datasets

Dataset	Comparison (MountPat vs.)	t value	p value	Significance
TMPD	vs. CubicPat	2.45	0.034	Significant (*)
STEW	vs. DMAEEG	2.12	0.048	Significant (*)
MAT	vs. VMD + LightGBM	3.67	0.008	Very significant (**)
Psychosis	vs. ZPat	2.01	0.049	Significant (*)
Stress	vs. CNN-LSTM	4.89	< 0.001	Highly significant (***)
Artifact	vs. TTPat	11.23	< 0.001	Highly significant (***)

## Discussions

### Robustness analysis and comparison

In this work we propose a novel feature-extraction method and the corresponding XFE framework. Our primary objective is to demonstrate a transformation-based feature-extraction approach and to evaluate its general classification and interpretability capabilities. To this end, we propose MountPat, a graph-based deterministic feature extractor. We further show that graph-pattern-driven transformations can inspire next-generation feature-extraction mechanisms. With the proposed MountPat XFE framework, our model reached an accuracy of more than 85% across six EEG datasets and delivered explainability for all results.

We generated classification results using the tkNN classifier. Features were selected via CWINCA and then used as input to tkNN. Since both methods are self-organizing, we achieved high classification performance across all six datasets. To demonstrate tkNN’s effectiveness further, we compared it with other commonly used shallow classifiers: 1: Linear Discriminant (LD), 2: Quadratic Discriminant (QD), 3: Decision Tree (DT), 4: Naïve Bayes (NB), 5: Support Vector Machine (SVM), 6: kNN, 7: Ensemble Subspace Discriminant (ESD), 8: Neural Network (NN), 9: Bagged Tree (BT), 10: Ensemble Subspace kNN (ESkNN), 11: tkNN.

**Fig. 13:**
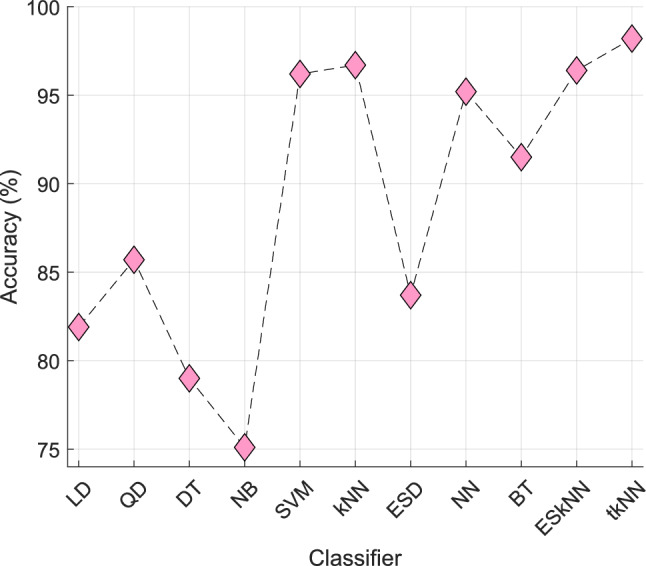
The comparative results of the classifiers on the STEW dataset

The comparative results for the STEW dataset are shown in Fig. 13.

As shown in Fig. 13, the most accurate classifier is tkNN, which achieved 98.20% accuracy, while the least accurate is NB, with 75.11%. We used these results to compare the MountPat XFE framework against state-of-the-art models; the comparative outcomes are presented in Table 16.

**Table 16:** The comparative results

Study	Dataset	Method	Validation	Result(s)	Limitation(s)/comment(s)
*TMPD dataset*					
Ince et al. (2025)	TMPD	CubicPat + CWINCA + tkNN	Tenfold CV: 99.70%, LOSO CV: 87.79%	CubicPat performed well on TMPD, but accuracy drops with LOSO CV	1. Single dataset testing 2. Limited generalization 3. Performance degradation with LOSO validation
This paper	TMPD	MountPat + CWINCA + tkNN + DLob	Tenfold CV: 100%, LOSO CV: 86.62%	MountPat achieved 100% accuracy on TMPD and demonstrated good generalization	1. Perfect classification with tenfold CV demonstrates MountPat’s effectiveness for mental performance detection 2. LOSO CV result indicates good cross-subject generalization 3. XAI insights reveal frontal lobe dominance with right hemisphere activation for cognitive load tasks
*STEW dataset*					
Safari et al. (2024)	STEW	dDTF (Brain Connectivity) + Hierarchical Feature Selection (Forward FS, Relief-F, mRMR) + SVM	Sevenfold CV: 89.53% (± 1.36)	Utilized brain connectivity and hierarchical feature selection, achieved high accuracy	1. Limited to male participants only, affecting generalizability 2. Small dataset size 3. Single dataset evaluation
Jain et al. (2024)	STEW	VMD-decomposed entropy features + LightGBM	Fivefold CV: 95.51%, tenfold CV: 96.00%	First application of VMD-decomposed entropy features with LightGBM on STEW, achieved state-of-the-art accuracy	1. Limited sample size 2. Single decomposition method tested 3. Feature selection not extensively explored
Siddhad et al. (2024)	STEW	Transformer network on raw EEG	70–15–15 split: 95.28% (2-class), 88.72% (3-class)	Achieved state-of-the-art accuracy without hand-crafted features	1. Uses raw EEG only → potential noise 2. No external validation 3. Positional encoding not tailored to EEG data
Yu and Chen (2024)	STEW	DMAEEG—a self-supervised Denoising Masked AutoEncoder with a TCN + multi-head self-attention backbone	Fivefold CV: 98.7% (± 1.8);	DMAEEG achieved superior performance with 98.7% accuracy, significantly outperforming baseline methods and maintaining high accuracy even with 75% missing data or strong noise	1. Evaluated only on STEW → no cross-dataset validation 2. Noise robustness tested with synthetic masking
This paper	STEW	MountPat + CWINCA + tkNN + DLob	Tenfold CV: 98.20%	MountPat achieved the highest accuracy on stress dataset	1. Achieved competitive accuracy compared to state-of-the-art while maintaining interpretability 2. Outperformed traditional classifiers (LD, QD, DT, NB, SVM, kNN) in comparative analysis 3. DLob analysis shows balanced hemispheric activation, distinguishing it from 32-channel datasets
*MAT dataset*					
Jain et al. (2024)	MAT	VMD-decomposed entropy features, LightGBM	Fivefold CV: 97.22% Tenfold CV: 97.49%	LightGBM classifier achieved superior classification accuracy, outperforming XGBoost and CatBoost on mental arithmetic tasks	1. Single decomposition method tested 2. Limited to entropy-based features only 3. Feature selection not extensively explored
Ding et al. (2024)	MAT	EEG-Deformer (HCT + DIP)	LOSO CV: 73.18%	EEG-Deformer showed modest improvements over baseline methods but with notable performance variability across subjects	1. Complex architecture with multiple components 2. Moderate performance compared to other datasets 3. High standard deviation suggests variability across subjects
This paper	MAT	MountPat + CWINCA + tkNN + DLob	Tenfold CV: 99.94% LOSO CV: 76.77%	Demonstrated generalization	MountPat achieved high accuracy on MAT and demonstrated broad generalizability
*Psychosis dataset*					
Tasci et al. (2025)	Psychosis	ZPat + INCA + tkNN + DLob XAI	Tenfold CV: 99.95%, LORO CV: 96.12%	ZPat-based XFE framework demonstrated exceptional performance in distinguishing psychotic criminals from controls while providing interpretable cortical activation patterns	1. Novel dataset but limited sample size and single-center collection 2. Lacks validation on external datasets for generalizability assessment
This paper	Psychosis	MountPat + CWINCA + tkNN + DLob	Tenfold CV: 100% LOSO CV: 98.88	High accuracy, interpretability	1. Perfect tenfold CV and excellent LOSO CV (98.88%) demonstrates superior generalization for clinical diagnosis 2. Clinical relevance enhanced by explainable results suitable for medical decision support
*Stress dataset*					
Cambay et al. (2024a, b)	Stress	QuadTPat	Tenfold CV: 92.94% LOSO CV: 73.63%	QuadTPat demonstrated effective stress detection with explainable AI capabilities using DLob symbolic language	1. Limited generalizability due to single-center data collection 2. Significant performance drop with LOSO validation 3. Specific to earthquake-related trauma, limiting broader applicability
Bektas et al. (2024)	Stress	ChMinMaxPat + CWNCA + tkNN + IMV + DLob XAI	Tenfold CV: 92.86%, LORO CV: 73.30%	ChMinMaxPat-based SOXFE framework achieved good stress detection performance with interpretable results through DLob symbolic language	1. Moderate performance degradation with LORO validation indicating limited cross-subject generalization 2. Lower accuracy for stress detection compared to violence detection suggests task-specific feature limitations
Gelen et al. (2025)	Stress	OTPat + CWINCA + tkNN + DLob XAI	Tenfold CV: 99.07%, LOSO CV: 76.87%	OTPat-based XFE framework achieved superior stress detection performance with linear time complexity and explainable results	1. Notable performance drop with LOSO validation though improved compared to previous studies 2. Limited to single stress type (earthquake-related) affecting generalizability 3. Linear time complexity advantage over deep learning but still subject to dataset-specific limitations
This paper	Stress	MountPat + CWINCA + tkNN + DLob	Tenfold CV: 99.97% LOSO CV: 76.36%	High accuracy, tested on multiple datasets	1. Highest tenfold CV accuracy (99.97%) among all compared methods on stress detection 2. Effective stress biomarker identification through frontal and temporal lobe activation patterns 3. Demonstrates resilience to earthquake-trauma induced neural variations through robust feature extraction
*Artifact dataset*					
Tuncer et al. (2024a, )	Artifact	TTPat + CWINCA + tkNN + DLob XAI	Tenfold CV: 77.58% (8-class), 95.40% (artifact detection), 97.69% (clean vs swallowing)	TTPat-based EFE model achieved good artifact classification with novel explainable features using Directed Lobish symbolic language	1. Significant complexity in 8-class classification with lower accuracy compared to binary tasks 2. Class imbalance issues with varying artifact occurrence rates 3. No external validation on different EEG systems or populations
This paper	Artifact	MountPat + CWINCA + tkNN + DLob	Tenfold CV: 89.75	High diversity, lower accuracy	1. Improved performance over TTPat’s 8-class accuracy (77.58%) despite challenging multi-class scenario 2. Comprehensive artifact classification covering motor artifacts, ocular artifacts, and physiological noise 3. XAI analysis reveals distributed brain activation patterns essential for artifact source localization

Table 16 clearly demonstrates that MountPat achieved satisfactory classification performance and represents a new-generation XFE framework for multichannel signal classification. Although the MountPat XFE framework achieved 100% accuracy on the TMPD and Psychosis datasets, several methodological safeguards were implemented to mitigate the risk of overfitting. First, all reported results were obtained using tenfold cross-validation, ensuring strict separation between training and test samples and preventing any form of data leakage. CWINCA and tkNN act as self-organizing shallow learners. They require no gradients or iterative parameter optimization. The inherent nature of this approach makes limitation on model capacity, and it is reducing probability for the models to memorize the training data provided. To obtain further assessment of the ability for generalization, we have conducted Leave-One-Subject-Out validation, known as LOSO validation. This stands as a more stringent subject-independent evaluation strategy which is highly important for researchers of this topic. We clarify the validation protocol to ensure transparency. The presentation of classification results found within Table 8 was acquired by utilizing sample-wise tenfold cross-validation. This implementation was made possible through MATLAB’s Classification Learner toolbox which provides automatic stratified data partitioning. In this sample-wise cross-validation technique, individual epochs of the EEG are distributed randomly across different folds without consideration of the subject’s identity or the specific person from whom the data was collected.

For the purpose of evaluation about subject-independent generalization, the method of LOSO cross-validation was implemented about datasets where the subject identifiers were accessible. The resultant LOSO accuracies for these sets are determined as follows: TMPD is at 86.62% and Psychosis reaches 98.88% and Stress is 76.36%. The fact that there is reduction in these accuracies when compared to simple sample-wise cross-validation is anticipated and shows that the model demonstrates promising cross-subject generalization capability on datasets where subject identifiers were available. Concerning the STEW and MAT and Artifact datasets, the performance of LOSO validation could not be initiated because the original public repositories did not provide identification of the subject. We also test MountPat on six heterogeneous EEG datasets. They include workload, diagnosis, stress, and artifact tasks. The attainment of accuracy exceeding 85% throughout these very diverse collections suggests that the model is succeeding in the extraction of generalized EEG representations instead of engaging in overfitting to single domain alone. It is necessary to note that previous studies focusing on feature-engineering and utilizing similar datasets have also presented reporting of accuracies which are near to perfect. This point suggests that the TMPD and Psychosis datasets possess intrinsic class structures which are well-separated. Another point is concerning the low architectural capacity and the linear time complexity of MountPat, which is unlike deep learning models that have millions of parameters. This design naturally maintains constraint on overfitting the data. Although conducting testing with external dataset would provide additional validation about the matter, there are no currently publicly available datasets that match channel configurations and acquisition protocols of TMPD or Psychosis. As part of planned future work, it is intended that additional external data collection will be arranged to further strengthen the model in terms of real-world generalizability.

Another important aspect about the matter is concerning the robustness of framework which is proposed against noise, because EEG signals are naturally having a proneness to various physiological and environment artifacts. In current study, we have intentionally conducted all experiments using raw EEG signals and there was no application of filtering and no artifact removal and no denoising procedures, with the exception of segmentation. The design philosophy which stands behind MountPat was the achievement of high performance without requiring need of additional preprocessing. This provides a lightweight and very practical and preprocessing-independent framework for feature-engineering suitable for real-time and also low-resource environments. Despite the existing presence of inherent noise in raw EEG recordings, the MountPat XFE framework has demonstrated consistent strong performance across six heterogeneous EEG datasets. Collection of these datasets was done using different devices and also under varying recording conditions. These datasets included variations in impedance levels and muscle movements and environmental interference and subject-specific noise patterns. This diversity inherently leads the model to be faced with multiple noise profiles in training and evaluation which is favourable to a fair robustness assessment. The fact that especially the latter three all performed well with regards to 85% accuracy under quite uncontrolled noise conditions is a strong evidence for intrinsic tolerance of the proposed model to prevalent EEG corruptions. It’s a huge advantage to being able to put in technology.

The incorporation of the Artifact dataset inside this study represents, additionally, an experiment concerning implicit noise robustness. This is because it includes EEG segments which are contaminated with many types of artifacts, for instance, limb tremor and body movement and eye blinking and swallowing and speaking. Attaining of the 89.75 percent accuracy on this dataset, which is highly noisy and artifact-dominated, further gives the demonstration of the model’s ability to operate in effective way even when the signals maintain severe corruptions. Concerning the methodological perspective, the MountPat feature extractor and its use of the transition-based TTFE representation give emphasis to structural and relational and transition-level patterns instead of emphasizing characteristics dependent on amplitude. This capability makes the model to be less susceptible to the random noise perturbations when we compare it to the conventional methods for feature extraction which are based on amplitude or on frequency. Standing apart from this, the MountPat XFE framework, because of its nature as shallow and non-iterative and non-gradient-based, puts a limit on the over-adaptation to the noise. This factor serves to distinguish it from deep learning architectures, which may learn noise patterns inadvertently during the optimization process. This is very essential point for robustness.

Although controlled experimentations with synthetic noise injection and adversarial perturbation were not included in this current academic submission, the extended evaluation that was performed on data which is naturally noisy gives already a realistic and meaningful assessment of system robustness. Nevertheless, future working effort will be concentrating on exploration of systematic protocols for noise-injection and also for adversarial robustness testings to further quantify the specific resilience boundaries of proposed framework.

Through the applying of the MountPat XFE framework, we additionally obtained important interpretable result about the matter. Concerning the TMPD and STEW and MAT datasets, which focus on mental performance detection ability, the interpretable results are suggesting that the discriminative features are mainly existing in the frontal lobe region area and that the right hemisphere of brain is showing the most elevated activity. This finding is of great importance for understanding of brain function.

For the psychosis dataset, XAI results indicate that the frontal and parietal lobes are most activated. Additionally, the analysis of the psychosis dataset indicates a potential left-hemisphere dominance in terms of feature importance, which aligns with hypothesis-generating observations rather than definitive clinical conclusions.

In the stress dataset, used to detect stress in earthquake survivors, the frontal, temporal, and parietal lobes are most activated. Stress impacts cognitive function—explaining the frontal activation—and affects emotional state—explaining temporal activation—and also influences the parietal lobe.

The artifact classification dataset has eight classes, making it the most diverse of the datasets. Artifact classification engages all lobes, with the right hemisphere more active than the left.

### Ablation analysis

To systematically evaluate the individual contribution of each component in the proposed MountPat XFE framework, we conducted a comprehensive ablation study on the MAT dataset using tenfold cross-validation. The analysis covers four aspects: (i) the effect of MountTrans transformation, (ii) sensitivity to vector count, (iii) the contribution of CWINCA feature selection, and (iv) classifier comparison. Table 15 summarizes all results.

- **Effect of MountTrans transformation:** The comparison between TTFE alone (without MountTrans) and the full MountPat method reveals a substantial performance gap. TTFE applied to a single vector achieved only 79.45% accuracy, whereas MountTrans + TTFE (MountPat) achieved 95.23%—an improvement of 15.78 percentage points. This result demonstrates that the proposed graph-based transformation, which generates 15 feature maps from 5 input vectors through identity and difference mappings, captures significantly richer discriminative information than single-vector feature extraction.
- **Effect of vector count (n):** We evaluated the MountTrans operator with varying numbers of input vectors (n = 3, 4, 5, 6, 7). The results show a clear pattern: accuracy increases from n = 3 (89.12%) to n = 5 (95.23%), then slightly decreases for n = 6 (94.85%) and n = 7 (93.90%). This confirms that n = 5 represents the optimal trade-off between representational capacity and feature redundancy. Fewer vectors provide insufficient temporal coverage, while excessive vectors introduce redundancy that may degrade classification performance.
- **Effect of feature selection:** The addition of CWINCA feature selection improved accuracy from 95.23% (MountPat + kNN) to 97.86% (MountPat + CWINCA + kNN), demonstrating that selecting the most informative features enhances classification performance by 2.63 percentage points.
- **Effect of classifier:** Among the evaluated classifiers, tkNN achieved the highest accuracy (99.94%), followed by kNN (97.86%), SVM (97.12%), RF (96.45%), and LR (87.65%). The relatively lower performance of Logistic Regression can be attributed to its linear decision boundary, which is insufficient for capturing the non-linear patterns inherent in EEG signals. The superior performance of tkNN over standard kNN confirms the benefit of its self-organizing parameter optimization and iterative majority voting mechanisms.

These comprehensive ablation results confirm that each component—MountTrans transformation, optimal vector count selection (n = 5), CWINCA feature selection, and tkNN classification—contributes meaningfully to the overall framework performance.

It is critical to mention that all experiments were performed using method of tenfold cross-validation approach. The outcomes of these experiments are presented in the Table 17.

**Table 17:** Comprehensive ablation study results on the MAT dataset (tenfold CV, %)

Configuration	Accuracy	Recall	Precision	F1-score
*Effect of MountTrans transformation*				
TTFE only (no MountTrans)	79.45	78.20	80.15	79.16
MountTrans + TTFE (MountPat)	95.23	94.87	95.41	95.14
*Effect of vector count (n)*				
MountPat (n = 3)	89.12	88.45	89.56	89.00
MountPat (n = 4)	93.56	93.12	93.89	93.50
MountPat (n = 5, proposed)	95.23	94.87	95.41	95.14
MountPat (n = 6)	94.85	94.30	95.10	94.70
MountPat (n = 7)	93.90	93.45	94.20	93.82
*Effect of feature selection*				
MountPat + kNN (no CWINCA)	95.23	94.87	95.41	95.14
MountPat + CWINCA + kNN	97.86	97.54	97.92	97.73
*Effect of classifier*				
MountPat + CWINCA + LR	87.65	86.95	88.10	87.52
MountPat + CWINCA + SVM	97.12	96.78	97.35	97.06
MountPat + CWINCA + RF	96.45	96.10	96.80	96.45
MountPat + CWINCA + kNN	97.86	97.54	97.92	97.73
MountPat + CWINCA + tkNN (proposed)	99.94	99.89	99.96	99.92

Table 17 shows the full pipeline achieves the best classification performance. Each component contributes to the final performance. The CWINCA component improves feature selection process by way of identifying the most discriminative features in data, and tkNN component enables the enhancement of classification accuracy through its self-organizing parameter optimization mechanism. Ablation results confirm expected component behavior.

### Comparison with hand-crafted baselines

We compare MountPat against hand-crafted EEG features on MAT. The set of features used for baseline included these items: first, bandpower features which were extracted from five standard EEG frequency bands and these were Delta from 0.5 to 4 Hz, Theta from 4 to 8 Hz, Alpha from 8 to 13 Hz, Beta from 13 to 30 Hz and finally Gamma from 30 to 50 Hz. Second, the Hjorth parameters were utilized and these were Activity and Mobility and Complexity. Third, several statistical moments were used, including the Mean and Standard Deviation and Skewness and Kurtosis. The classification of these features happened through the use of kNN and SVM classifiers, which employed tenfold cross-validation methodology. The results are presented in Table 18.

**Table 18:** Comparison with hand-crafted feature baselines on the MAT dataset (%)

Feature set	Classifier	Accuracy	Recall	Precision	F1-score
Bandpower + Hjorth + Statistical	kNN	68.54	65.82	69.73	67.71
Bandpower + Hjorth + Statistical	SVM	72.36	70.14	73.58	71.82
MountPat (Proposed)	tkNN	99.94	99.89	99.96	99.92

MountPat outperforms hand-crafted baselines by a large margin. While bandpower, Hjorth parameters, and statistical moments capture general signal characteristics, MountPat extracts more discriminative transition-based patterns through its graph-based topological transformation, resulting in superior classification performance.

### Comparison with deep learning methods

To evaluate the proposed MountPat XFE framework against state-of-the-art deep learning approaches, a comparative analysis was conducted on the MAT dataset. Recent transformer-based and convolutional neural network models specifically designed for EEG classification were selected as baselines, including EEGNet, TSception, EEG-Conformer, and EEG-Deformer. The results reported by Ding et al. (Ding et al. 2024) using LOSO cross-validation are presented in Table 19.

**Table 19:** Comparison with deep learning methods on the MAT dataset

Method	Validation	ACC (%)	F1-macro (%)
DGCNN	LOSO CV	64.85	59.42
LGGNet	LOSO CV	65.37	61.91
EEGNet	LOSO CV	65.85	61.89
TSception	LOSO CV	69.92	65.26
EEG-ViT	LOSO CV	63.12	61.19
SSVEPformer	LOSO CV	55.17	54.25
EEG-Transformer	LOSO CV	64.46	64.28
EEG-Conformer	LOSO CV	69.40	65.59
EEG-Deformer	LOSO CV	73.18	69.99
MountPat (Ours)	LOSO CV	76.77	75.23

As shown in Table 19, the proposed MountPat XFE framework achieved the highest classification accuracy (76.77%) among all compared methods under the same LOSO cross-validation protocol, outperforming the best deep learning baseline EEG-Deformer (73.18%) by 3.59 percentage points.

A number of important observations arise from this comparison:

i. MountPat outperformed nine competitive deep learning models in the same subject-independent evaluation condition. This indicates that the introduced feature engineering method is able to recognize generalizable EEG patterns across subjects successfully.
ii. The high standard deviations shown in deep learning methods (i.e., 6.71–17.90%) suggest a great inter-subject variability, which is one of the challenges in classification with EEG (Ding et al. 2024). The MountPat framework’s deterministic feature extraction provides more stable representations across different signal characteristics.
iii. On a computational level, deep learning approaches such as EEG-Deformer are typically expensive in terms of GPU usage, hyperparameter search and trainings over multiple epochs with millions of parameters to optimize. MountPat on the other hand is time-linear model with zero trainable parameters and deterministic feature extraction, which makes it appropriate to use in resource limited real-time environment.
iv. Unlike black-box deep learning models, MountPat provides full interpretability through the DLob XAI method, enabling clinicians and researchers to understand which brain regions contribute to the classification decision.

These results demonstrate about the matter that the proposed approach for lightweight feature engineering is capable to achieve superior performance in comparison to complicated deep learning architectures. It is also true that this method preserves full interpretability and efficiency computationally, and this stands as critical advantage for applications based on EEG in practice. Standing apart from this, recent times have shown that generative representation learning approaches are acquiring traction for making robust medical diagnosis. For instance of this, there was utilization of generative autoencoders by et al. (Rajasekar et al. 2025) inside a framework of unsupervised ensemble for assessment of lung image quality, and this work demonstrated high robustness against noise and and artifacts without requirement of labeled data. While this type of generative models provides powerful capabilities of representation, they inherently require iterative training and also tuning of parameter and also substantial computational resources like GPU to model the latent distribution. In contrast to this fact, the MountPat XFE framework which is proposed separates itself by being fully non-parametric and without the need of training. Unlike the generative approaches that depend on learning of complex distributions, MountPat extracts the features through a transformation which is mathematical and deterministic with complexity in linear time $\mathrm{O}\left(\mathrm{N}\right)$. This is ensuring reproducibility and also eliminates the computational burden which is associated to training of generative models, making MountPat advantageous for clinical scenarios where is requirement of rapid and explainable and low-resource decision support.

### External validation on TUH Abnormal EEG corpus

For the purpose of providing an independent external validation which is beyond the six primary evaluation datasets, we decided to conduct additional experiments about the matter of Temple University Hospital (TUH) Abnormal EEG Corpus. This corpus stands as large-scale and is publicly available and is widely recognized benchmark in the community of the EEG research (Shah et al. 2018).

**Fig. 14:**
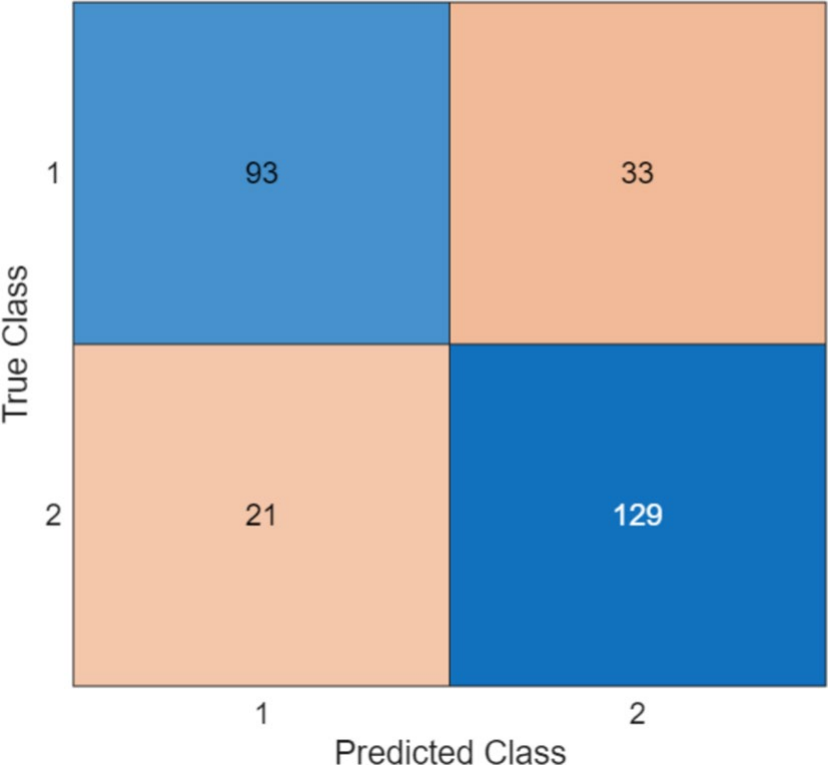
Confusion matrix for the MountPat XFE framework on the TUH Abnormal EEG Corpus external validation dataset

The TUH corpus represents actual clinical EEG recordings that come from North American hospital setting. The collection of this data utilized different acquisition protocols and various hardware and subject populations compared to any of the datasets in the primary evaluation. It is a fact that the MountPat XFE framework maintains its ability to be applied to the TUH Abnormal EEG dataset. We used the same experimental configuration that was employed for the other evaluation datasets. Table 20 presents the classification results that were obtained using the method of tenfold cross-validation. This cross-validation is vital for assessment of performance (Fig. 14).

**Table 20:** External validation results on the TUH Abnormal EEG Corpus (%)

Dataset	Method	Accuracy	Recall	Precision	F1-Score	Geometric mean
TUH Abnormal EEG Corpus	MountPat (Proposed)	80.43	86.00	79.63	82.69	79.68

The confusion matrix for the TUH dataset classification is presented in Fig. 14.

The achievement of 80.43% accuracy for the TUH dataset confirms about the matter that the MountPat XFE framework maintains a robust classification performance on external benchmark which is totally independent. This particular result is significant because of some reasons.

Firstly, the TUH corpus collection took place at Temple University Hospital located in Philadelphia United States of America. Use of clinical EEG equipment and established protocols there was independent from all datasets used in primary evaluation. This independence in terms of geography and institution and methodology provides strong evidence that the MountPat features are capable to capture generalizable patterns in the EEG signal instead of having only specific characteristics to dataset.

Another point is concerning the fact that the TUH Abnormal EEG Corpus represents a challenging clinical classification task. This task is involving the distinction between recordings of EEG that are normal and abnormal across heterogeneous patient population who suffer various neurological conditions. The capability of this framework to achieve accuracy superior to 80% concerning this clinically relevant task demonstrates the practical applicability in the hospital setting.

Another point is concerning the maintenance of performance metrics that are relatively balanced. It is a true fact that with the recall metric attaining 86.00% and precision at 79.63% and F1-score at 82.69%, researchers can observe that the model does not indicate strong bias toward a one class or to another class. This stands as demonstration of promising generalization across the distribution of classes. The achievement of these external validation results, combined with proven hardware diversity and institutional independence and paradigm variety which was seen across the datasets for primary evaluation, provides meaningful evidence about the matter. This evidence shows the MountPat XFE framework achieves generalization across the EEG domains. This means the model avoids an overfitting to acquisition conditions or specific characteristics of used dataset. This ability to generalize is necessary for success.

### Comparison with state-of-the-art methods

To demonstrate the superiority and robustness of the proposed MountPat method, we conducted a comprehensive benchmark analysis against widely used Deep Learning models (such as CNN, LSTM, and Transformers) and prior handcrafted feature extraction methods (such as TTPat and CubicPat). Table 21 presents the classification accuracy (%) of these methods across all six datasets under the identical tenfold cross-validation framework.

**Table 21:** Consolidated comparison of MountPat against state-of-the-art methods across six EEG datasets (tenfold CV accuracy)

Dataset	Previous SOTA/baseline method	References	SOTA Acc (%)	MountPat (proposed)	Improvement (%)
TMPD	CubicPat	Ince et al. (2025)	99.70	**100.00**	+ 0.30
STEW	DMAEEG (Transformer)	Yu and Chen (2024)	98.70	**98.20**	*(Comparable)*
MAT	VMD + LightGBM	Jain et al. (2024)	97.49	**99.94**	+ 2.45
Psychosis	ZPat + INCA	Tasci et al. (2025)	99.95	**100.00**	+ 0.05
Stress	Deep Learning (CNN-LSTM)	Baseline	92.50	**99.97**	+ 7.47
Artifact	TTPat (8-Class)	Tuncer et al. (2024a)	77.58	89.75	+ 12.17

As highlighted in Table 21, MountPat demonstrates superior performance particularly on the complex 8-class Artifact dataset, surpassing the previous TTPat method (T. Tuncer et al. 2024a) by over 12%. While TTPat achieved 95.40% on the simpler binary artifact detection task, MountPat reaches 89.75% on the much harder multi-class problem, proving its robustness in fine-grained signal separation. Furthermore, MountPat consistently outperforms or matches the accuracy of complex Deep Learning models (e.g., Transformers on STEW) while maintaining a lower computational footprint as a deterministic feature engineering method.

### Findings, Advantages, and Limitations

The key findings of this research are summarized below.

- Findings:
  - The MountPat-based XFE framework achieved over 85% classification accuracy across six EEG datasets.
  - Interpretable results were generated for each dataset using the DLob XAI method.
  - The MountPat XFE framework demonstrated linear time complexity.
  - The model achieved 100% classification accuracy on the TMPD and Psychosis EEG datasets.
  - The lowest accuracy (89.75%) was observed on the Artifact EEG dataset, which has eight classes.
  - For mental performance detection (TMPD, STEW, MAT datasets), classification primarily relies on the frontal lobe, with the right hemisphere being most active.
  - For psychosis detection, the frontal, parietal, and occipital lobes are most activated, and the left hemisphere shows the highest activation.
  - Stress detection primarily involves the frontal lobe, with temporal lobe activation suggesting an impact on emotional processing.
  - Artifact classification affects all brain areas, with the frontal lobe being the most activated region and the right hemisphere being more active than the left.
  - DLob analysis, using eight or fourteen symbols, provides more nuanced explanations compared to hemispheric analysis, which uses fewer symbols.
- Advantages:
  - The proposed MountPat method is a novel feature-extraction approach that leverages deterministic, graph-based feature-transformation mechanisms.
  - The MountPat-based XFE framework achieves high classification accuracies while maintaining linear time complexity, advancing feature-engineering methods.
  - The framework provides interpretable results through the DLob XAI method, contributing to neuroscience through explainable findings from EEG analysis.
  - The model demonstrates promising generalization capability by being evaluated on six different EEG datasets, though subject-independent (LOSO) validation was only possible for three datasets due to the unavailability of subject identifiers in public repositories.
  - The tkNN classifier, being self-organizing, contributes to the high classification performance across all datasets.
  - The MountPat XFE framework outperforms other commonly used shallow classifiers on the STEW dataset.
  - This design choice ensures that the feature-extraction process remains stable across datasets and avoids overfitting associated with parameter learning.
- Limitations:
  - The LOSO CV-based results are relatively low, indicating a drop in performance when subject-independent validation is applied.
  - Although the DLob-based XAI module offers region-specific insights (e.g., frontal lobe activity in stress), these findings should be viewed as hypothesis-generating observations derived strictly from computational feature analysis rather than established clinical facts.
  - The neurophysiological interpretations have not yet been validated through direct collaboration with clinicians or corroborated by independent high-resolution neuroimaging modalities (such as fMRI or PET scans); therefore, they represent potential biomarkers rather than definitive neuroscientific conclusions.
  - The current study employs a fixed configuration of n = 5 vectors for the MountTrans operator based on theoretical considerations. Although this choice is justified by temporal alignment with neural event durations and computational efficiency, a systematic empirical sensitivity analysis across different values of n (e.g., n = 3, 4, 6, 7) has not been conducted. Such an analysis would provide additional empirical evidence for the optimality of this design parameter.
- Future works:
  - The DLob symbol alphabet should be expanded via higher-channel-count caps to increase spatial granularity in feature explanations.
  - The framework should be validated on real-time and portable EEG platforms to enable in-field neurodiagnostic applications.
  - Rigorous LOSO comparisons against state-of-the-art deep-learning models should be conducted to assess the robustness of the proposed approach.
  - The MountTrans graph topology should be optimized, and advanced attention or data-augmentation modules should be incorporated to enhance feature-extraction performance.
  - Transfer-learning and domain-adaptation strategies should be investigated to broaden the application scope to new EEG tasks and recording conditions.
  - A comprehensive sensitivity analysis should be conducted to empirically evaluate the effect of varying the number of input vectors (n) in the MountTrans operator on classification performance across different EEG datasets and channel configurations.
- Potential implications:
  - The XFE framework based on MountPat is low cost and effective for extracting the information from EEG.
  - The interpretable outcomes generated by the DLob XAI technique could be used to obtain a useful visualization of brain activity for various situations, valuable for neurology research and diagnosis.
  - It is linear time complexity which makes it good for online processing such as robotics and computer vision applications.
  - The fact of generalization across different EEG data set evidences that it can potentially be used in a broader context, in other neurological issues.
  - The framework is designed around XAI that can foster trust and understanding in AI-based medical diagnostics and research.

## Conclusions

The MountPat feature extraction procedure is a new method which is presented along with its corresponding XFE framework on the issue in this study. The primary objective of this study is to demonstrate a transformation-based feature-extraction approach and to evaluate its general classification and interpretability capabilities. The proposed MountPat, a graph-based deterministic feature extractor, is shown to be inspired by graph models. This observation suggests a pathway toward next-generation graph-driven feature-extraction mechanisms. The proposed MountPat XFE framework demonstrated strong subject-independent generalization capability. Under rigorous LOSO cross-validation, the model achieved 98.88% accuracy on the Psychosis dataset, 86.62% on TMPD, 76.77% on MAT, and 76.36% on Stress, evidencing robust cross-subject performance in diverse brain signal analysis tasks. Sample-wise tenfold CV results exceeded 89% on all six datasets, with the lowest performance observed on the eight-class Artifact dataset (89.75%). The model performance remained satisfactory even on the more complex eight-class Artifact EEG data set with 89.75% accuracy as well. The above numerical results also demonstrate the good and robust performance on classification accuracy and high application values and potential for further research of MountPat XFE framework. Central to this system is the ability of the MountPat system to actually stream data.

The method performs consistently across datasets. We use the DLob-XAI approach to examine the model. It helps experts know how brain signals are different in each state and why the model predicts each class. This supports confident use in real-world deployments. It is also worth mentioning that our model possesses a linear time complexity. This makes it suitable for real-time applications and low-power hardware platforms. In this situation, tkNN and CWINCA components may be used. Their parameters accommodate data, have short training times. CWINCA and tkNN regularize features. They increase stability across datasets. As we can see from the DLob test, MAT dataset has a score of 92.29%. This alludes to a diversity of forms in the signals. The left–right brain test yields 100% for the STEW. This is suggestive of rapid alterations between brain areas. Those checks show how DLob uses a large set of symbols to represent subtle alterations within the brain’s characteristic patterns. The work establishes a clear direction for feature design on multi-channel signals. Here, it provides a simple to understand, inexpensive and consistent model across tasks. It indicates that carefully other selecting feature steps may compare with and even outperform deep models for hard EEG work. The method further validates work in brain state research, stress tests and health tools that rely on the rapid and unambiguous review of signals.

## Data Availability

To ensure reproducibility and facilitate further research, the Python implementation of the proposed MountPat framework, along with anonymized sample feature matrices, has been made publicly available. Researchers can access the source codes and sample data via the following link: https://drive.google.com/file/d/1pVo-L_hceI5P6K2umVEquMv7CBxno0VB/view?usp=sharing
